# Mass Spectrometric Evaluation of β-Cyclodextrins as Potential Hosts for Titanocene Dichloride

**DOI:** 10.3390/ijms22189789

**Published:** 2021-09-10

**Authors:** Pia S. Bruni, Stefan Schürch

**Affiliations:** Department of Chemistry, Biochemistry and Pharmaceutical Sciences, University of Bern, 3012 Bern, Switzerland; pia.bruni@unibe.ch

**Keywords:** metallocene, gas-phase reaction, cyclodextrin, mass spectrometry, host-guest complex

## Abstract

Bent metallocene dichlorides (Cp_2_MCl_2_, M = Ti, Mo, Nb, …) have found interest as anti-cancer drugs in order to overcome the drawbacks associated with platinum-based therapeutics. However, they suffer from poor hydrolytic stability at physiological pH. A promising approach to improve their hydrolytic stability is the formation of host-guest complexes with macrocyclic structures, such as cyclodextrins. In this work, we utilized nanoelectrospray ionization tandem mass spectrometry to probe the interaction of titanocene dichloride with β-cyclodextrin. Unlike the non-covalent binding of phenylalanine and oxaliplatin to β-cyclodextrin, the mixture of titanocene and β-cyclodextrin led to signals assigned as [βCD + Cp_2_Ti–H]^+^, indicating a covalent character of the interaction. This finding is supported by titanated cyclodextrin fragment ions occurring from collisional activation. Employing di- and trimethylated β-cyclodextrins as hosts enabled the elucidation of the influence of the cyclodextrin hydroxy groups on the interaction with guest structures. Masking of the hydroxy groups was found to impair the covalent interaction and enabling the encapsulation of the guest structure within the hydrophobic cavity of the cyclodextrin. Findings are further supported by breakdown curves obtained by gas-phase dissociation of the various complexes.

## 1. Introduction

Good solubility and stability in a physiological aqueous environment are essential prerequisites of a pharmaceutically active compound in order to develop its therapeutic activity. Within this context, low bioavailability due to high hydrophobicity, fast degradation, or deactivation as a result of rapid reaction with unspecific targets represent a continuous challenge in drug formulation [1,2]. Organometallic anti-cancer drugs based on transition metal complexes, such as cisplatin and its analogs and bent metallocene complexes, which are known to target nucleic acids as well as proteins, are severely suffering these difficulties [3].

**Figure 1:**
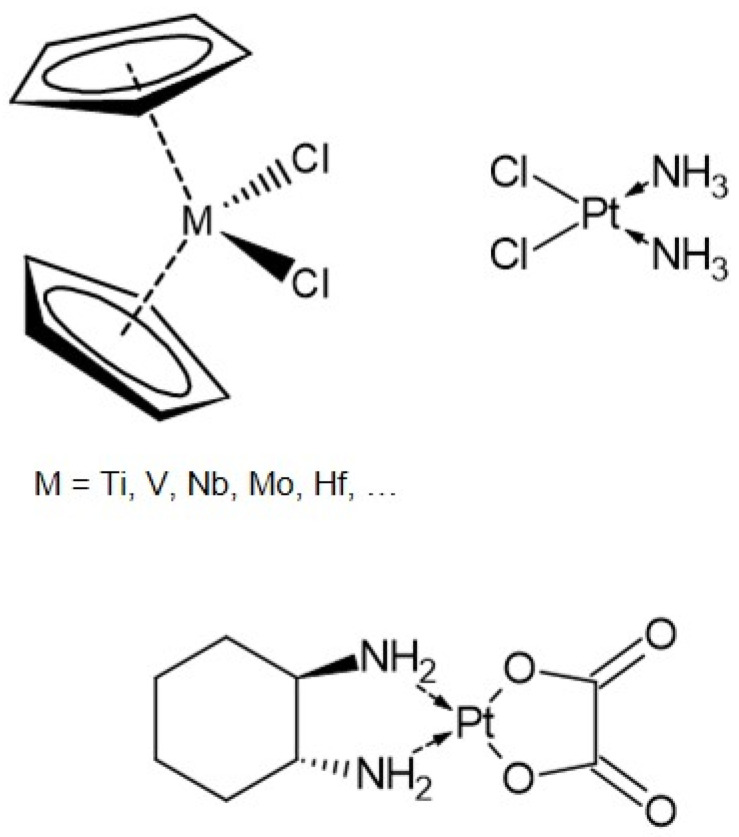
Structure of bent metallocene dichlorides (M = Ti, V, Nb, Mo, Hf, …), cisplatin, and its derivative oxaliplatin.

Cisplatin exhibits a planar structure with a platinum(II) coordination center surrounded by two ammine and two chloride ligands in cis configuration (Figure 1) [4]. It is widely used in cancer treatment but suffers from severe side-effects due to its toxicity and accruing drug resistance [5]. By alteration of the ligands, alternative drugs (e.g., carboplatin, oxaliplatin) were developed to overcome these drawbacks [4]. In addition, transition metal centered bent metallocenes emerged as a promising alternative to platinum-based drugs [5].

### 1.1. Bent Metallocenes

Bent metallocene dichlorides (Cp_2_MCl_2_) are built of a transition metal center M, often Ti, Mo, Nb, or V, two η^5^-cyclopentadienyl ligands (Cp) and two chloride ligands (Figure 1) [3,6,7]. In contrast to the platinum-based compounds, the transition metal is generally in the +IV oxidation state, and the overall structure resembles a distorted tetrahedron [6]. Among these metallocenes, titanocene had entered clinical trials in the 1990s, but was finally rejected due to insufficient response to metastatic cancers [8,9]. Nevertheless, the proven antiproliferative activity of metallocenes encourages further investigation of their interaction with potential biological targets.

A key step for the development of the anti-cancer activity of these compounds is the exchange of the chloride ligands for hydroxy ions in an aqueous environment [4,10]. Hydrolysis of the first chloride ligand occurs rapidly, whereas the exchange of the second chloride ligand is dependent on the metal species and the pH of the solution. For titanocene dichloride, the half-life of the second chloride ligand is ~50 min [11]. In consequence of this hydrolysis, the pH of the solution drops significantly, resulting in substantial difficulties in the application in biological systems due to increased side effects [6]. In the case of titanocene, raising the pH to physiological conditions leads to hydrolysis of the two Cp-ligands and the formation of insoluble titanium oxide species [6,7,12]. In contrast, molybdenocene shows increased hydrolytic stability of the Cp-ligands at higher pH [3,13]. Although some metallocene dichlorides exhibit higher stability than others, their aqueous stability is generally poor and represents a major challenge for their administration and necessitates the processing of the drug prior to usage [5,7]. One approach is the replacement of one or both chloride ligands by different halogenides or organic ligands [6,14,15], while other studies aimed at a more targeted drug delivery by functionalization of the Cp-ligands (e.g., titanocene Y) or alteration of the metal center [5,6,7,14,15,16,17,18].

Another approach to prevent metallocenes from extensive hydrolysis in an aqueous environment is the encapsulation within a host molecule [12,19]. Additional advantages of this approach are the increased aqueous solubility and bioavailability and a lowered toxicity [1,4,12,13,15,20,21]. Host molecules typically combine a rather hydrophobic inner cavity, which undergoes non-covalent interaction with the guest molecules (or parts of it) with a more polar outer surface that promotes better solubility in an aqueous environment [1,22,23]. Examples of host molecules successfully applied in drug delivery are cyclodextrins, cucurbiturils, pillarenes, and calixarenes [24,25].

### 1.2. Cyclodextrins as Host Molecules

**Figure 2:**
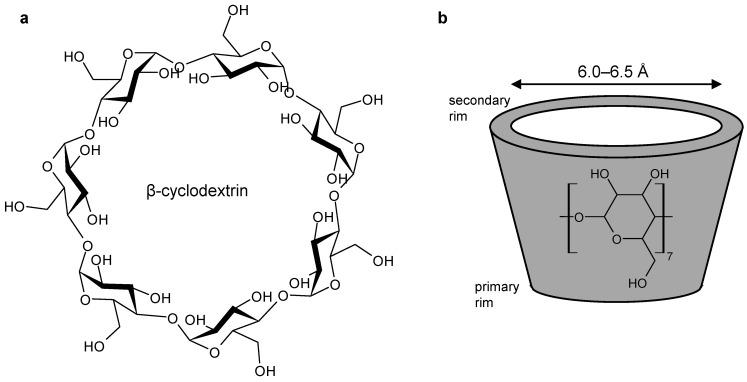
(**a**) Structural formula of β-cyclodextrin; (**b**) sketch of the truncated cone formed by β-cyclodextrin with the size of the cavity.

Due to their low toxicity [1,23,26], cyclodextrins are considered safe excipients when administered orally. In addition, they have found widespread applications as hosts of poorly soluble therapeutic agents, as summarized in recent review articles [2,22,23,26,27]. Cyclodextrins are a family of cyclic oligosaccharides composed of (1,4)-linked α-D-glucopyranose units. The naturally occurring α-, β-, and γ-cyclodextrins, comprising 6, 7, or 8 subunits, respectively, are the result of the enzymatic degradation of starch. Due to the chair conformation of the glucopyranose monomer, cyclodextrins adopt the shape of a truncated cone, with the hydroxy groups located at the rim and the skeletal carbon atoms providing the hydrophobic character of the cavity (Figure 2) [1,2,22,23,26,27,28,29,30]. The ability to form a host-guest system depends on various factors. The size of the cavity of the host molecule has to fit the size of the guest structure. In the case of bent metallocenes, the cavity size of β-cyclodextrin (6.0–6.5 Å) is suitable for accommodating the Cp-ligands of titanocene dichloride with a diameter of 5.8 Å [1,22,24]. The driving forces of the interaction between the host and guest structures include hydrophobic interactions, electrostatic, van der Waals, as well as hydrogen bonding [1,2,25,30,31]. A key criterion that defines host-guest complexes is that no covalent bonds are broken nor newly formed [22,23,25,26]. Consequently, the host-guest complex is in equilibrium with the free cyclodextrin and its guest molecules in solution [23,26,27].

The formation of hydrogen bonds between adjacent hydroxy groups at the rim of cyclodextrins leads to a rather rigid structure and lowers their solubility, as the interaction with the surrounding water is decreased [22]. By modification of the hydroxy groups, e.g., by conversion into methyl-, hydroxypropyl-, sulfobutyl ether-, or acetyl-moieties, the regular hydrogen-bonding within the natural cyclodextrins is disrupted, thus, enabling better interaction with the surrounding water molecules and increasing the solubility [2,22,26,27].

### 1.3. Investigation of Complexes

Common techniques used to investigate the stability of cyclodextrin-substrate complexes are calorimetric analysis, nuclear magnetic resonance spectroscopy (NMR), X-ray diffraction, fluorescence spectroscopy, and FTIR. Phase solubility studies and conductometry titration are alternative methods suitable for the investigation of the efficiency of cyclodextrin complexation [2,13,26,31,32]. Furthermore, theoretical approaches and computational modeling have been applied to predict the structure of complexes [4,33].

Using these methods, the encapsulation of organic molecules including amino acids [2,31,34] and platinum compounds [4,35] has been described, and evidence for the inclusion of bent metallocenes with confirmed antitumor activity (M = Ti, Mo, Nb, V) in cyclodextrins was provided by various authors [12,13,15,29,33,36,37,38,39]. Depending on the metallocene, its orientation and hydrolysis state for encapsulation was found to be different. Based on an experimental and theoretical study, Braga et al. suggested molybdenocene dichloride entering the β-cyclodextrin cavity preferably with only one Cp-ligand [33], whereas Morales et al. proposed niobocene to be incorporated as Cp_2_NbCl_2_OH, with the inclusion of one or both Cp-ligands occurring [12]. Theoretical considerations by Riviş et al. led to the conclusion that the formation of titanocene/cyclodextrin inclusion compounds is feasible and enhances the cytotoxic activity of titanocene as it enables controlled release of the drug and diminishes the hydrolysis of ligands [19].

### 1.4. Mass Spectrometry

Although inclusion complexes with cyclodextrins have been studied using various techniques, mass spectrometry contributed only rarely. With soft-ionization techniques such as matrix-assisted laser desorption/ionization and electrospray ionization, mass spectrometry has become a useful tool for the elucidation of molecular weights, stoichiometries, and even non-covalent interactions within supramolecular assemblies [25,32,34,39,40]. Mass spectrometry may provide advantages over different analytical techniques in terms of sensitivity and speed [32], and tandem mass spectrometric experiments employing collisional activation have successfully been applied to the evaluation of the relative stabilities of host-guest complexes [25]. The technique has been applied to provide information on the encapsulation of organic structures [25,34,41], ferrocene and its derivatives [39], as well as bent metallocenes [38] in cyclodextrins.

Mass spectrometric data on the inclusion of bent metallocenes have been published only sparsely. The scope of this investigation is to provide further insight into the interaction of titanocene dichloride with β-cyclodextrin and methylated cyclodextrin derivatives to assess the potential of these carbohydrate macrocycles as excipients in the formulation of bent metallocene-based drugs.

## 2. Results

Despite electrospray being a soft ionization technique, the decomposition of analyte ions due to collision with residual gas in the interface region may occur and potentially compromise the results. To probe the extent of such interfering effects and to demonstrate the capability of mass spectrometry to visualize host-guest interactions of cyclodextrin with organic compounds as well as transition metal complexes, phenylalanine, and the anti-cancer agent oxaliplatin, were chosen as guests. In a second step, the study was extended to the interaction of cyclodextrin with the bent metallocene titanocene dichloride.

Initial experiments aimed at the identification of peaks originating from the individual host and guest molecules, as besides the generation of molecular ions, adduct formation and in-source decomposition were expected, resulting in rather complex mass spectra. Based on high-resolution accurate mass analysis with deviations in the low parts per million (ppm) range and the detected isotopic pattern, the elemental composition of molecular as well as fragment ions can be determined. The full scan mass spectrum of β-cyclodextrin shows the protonated species [βCD + H]^+^ (*m*/*z* 1135.3798, 2.5 ppm), the ammonium adduct [βCD + NH_4_]^+^ (*m*/*z* 1152.4060, 2.1 ppm), and the alkali metal-adducts [βCD + Na]^+^ (*m*/*z* 1157.3599, 0.8 ppm) and [βCD + K]^+^ (*m*/*z* 1173.3340, 0.9 ppm). Furthermore, in-source fragmentation products due to the loss of glucopyranose subunits [glc_n_ + H]^+^ (n = 2–6; *m*/*z* 325.1128, −0.3 ppm; *m*/*z* 487.1653, −0.8 ppm; *m*/*z* 649.2186, 0.0 ppm; *m*/*z* 811.2720, 0.7 ppm; *m*/*z* 973.3255, 1.3 ppm) were identified (Supplementary materials Table S1) [42].

Electrospray ionization mass spectrometry of guest species primarily results in their protonation and the formation of the corresponding sodium adducts. Additionally, fragment ions originating from in-source decomposition were detected. In the case of phenylalanine, the protonated form [Phe + H]^+^ (*m*/*z* 166.0856, −4.8 ppm), the alkali adducts [Phe + Na]^+^ (*m*/*z* 188.0676, −3.2 ppm) and [Phe + K]^+^ (*m*/*z* 204.0416, −2.5 ppm), as well as the immonium ion [Imm(Phe)]^+^ (*m*/*z* 120.0803, −4.2 ppm) were identified (Supplementary materials Table S2). Comparable signals appeared for oxaliplatin, where [oxaliPt + H]^+^ (*m*/*z* 395.0695, 5.3 ppm) and [oxaliPt + Na]^+^ (*m*/*z* 420.0514, 4.8 ppm) were detected (Supplementary materials Table S3). The mass spectra of titanocene dichloride show signals corresponding to [Cp_2_TiCl]^+^ (*m*/*z* 212.9940, −2.3 ppm) and the hydrolysis products [Cp_2_Ti(OH)]^+^ (*m*/*z* 195.0278, −3.1 ppm), [CpTi(OH)_2_ + H_2_O]^+^ (*m*/*z* 165.0021, −3.0 ppm), [CpTi(OH)_2_]^+^ (*m*/*z* 146.9915, −3.4 ppm), and [Ti(OH)_3_ + H_2_O]^+^ (*m*/*z* 116.9657, −4.3 ppm), as well as [Cp_2_Ti(COOH)]^+^ (*m*/*z* 223.0227, −2.7 ppm), which was formed due to the presence of formic acid (Supplementary materials Table S4). Though the samples were subjected to mass spectrometric analysis immediately after getting in contact with water, rapid hydrolysis of the chloride ligands could not be prevented. Experiments performed in pure MeOH resulted in comparable peaks, with the difference that complexes comprising not only hydroxo-, but also methoxo-ligands, such as [CpTi(OH)(OMe)(H_2_O)]^+^ at *m*/*z* 179.0178 (−2.2 ppm), were detected as well (Supplementary materials Table S5).

### 2.1. Phenylalanine and Oxaliplatin Complexes

Beside the previously described ions, analysis of mixtures of β-cyclodextrin with phenylalanine and oxaliplatin revealed signals referring to the protonated inclusion complexes [βCD + Phe + H]^+^ (*m*/*z* 1300.4575, 1.2 ppm) and [βCD + oxaliPt + H]^+^ (*m*/*z* 1532.4389, 1.1 ppm), as well as the sodium adducts [βCD + Phe + Na]^+^ (*m*/*z* 1322.4392, 0.9 ppm) and [βCD + oxaliPt + Na]^+^ (*m*/*z* 1554.4207, 1.0 ppm), respectively (Supplementary materials Tables S6 and S8).

**Figure 3:**
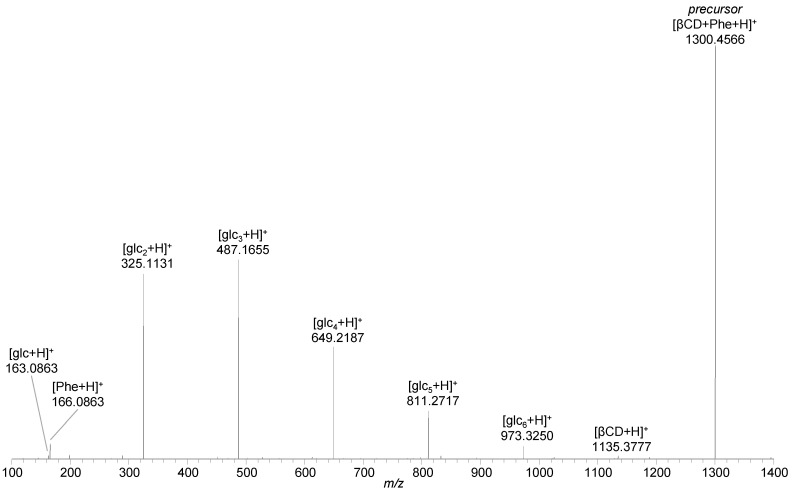
Higher energy collision induced dissociation (HCD) spectrum of [βCD + Phe + H]^+^ (*m*/*z* 1300.48) at 37 eV (16% normalized collision energy (NCE)) showing the separation of the precursor ion into [Phe + H]^+^, [βCD + H]^+^, and β-cyclodextrin fragment ions [glc_n_ + H]^+^ (n = 1–6).

A higher energy collision induced dissociation (HCD) experiment with [βCD + Phe + H]^+^ as the precursor ion gave rise to signals referring to protonated phenylalanine [Phe + H]^+^ (*m*/*z* 166.0863, 0.0 ppm), protonated β-cyclodextrin [βCD + H]^+^ (*m*/*z* 1135.3777, 0.6 ppm), and protonated cyclodextrin fragments [glc_n_ + H]^+^ (n = 1–6; *m*/*z* 163.0601, 0.0 ppm; *m*/*z* 325.1131, 0.6 ppm; *m*/*z* 487.1655, −0.4 ppm; *m*/*z* 649.2187, 0.2 ppm; *m*/*z* 811.2717, 0.4 ppm; *m*/*z* 973.3250, 0.8 ppm), indicating complete separation of host and guest along with the simultaneous decomposition of the host structure (Figure 3, Supplementary materials Table S7). Fragmentation of the oxaliplatin inclusion complex [βCD + oxaliPt + H]^+^ resulted in abundant signals of protonated oxaliplatin [oxaliPt + H]^+^ (*m*/*z* 398.0681, 1.8 ppm) and protonated cyclodextrin fragments still interacting with oxaliplatin [glc_n_ + oxaliPt + H]^+^ (n = 3–6; *m*/*z* 884.2279, 2.3 ppm; *m*/*z* 1046.2812, 2.4 ppm; *m*/*z* 1208.3344, 2.4 ppm; *m*/*z* 1370.3872, 2.1 ppm), whereas fragments of β-cyclodextrin without oxaliplatin were of very low intensity (Supplementary materials Table S9). The molecular formula determined by accurate mass analysis of this singly charged ion hints at neutral β-cyclodextrin fragments interacting with neutral oxaliplatin in a non-covalent manner with the positive charge provided by an additional proton, whose exact location is not defined. Despite the fact that separation of the host and guest structures was not detected as for phenylalanine, the non-covalent interaction between oxaliplatin and the macrocycle indicates the formation of an inclusion complex.

### 2.2. Titanocene Complex

**Figure 4:**
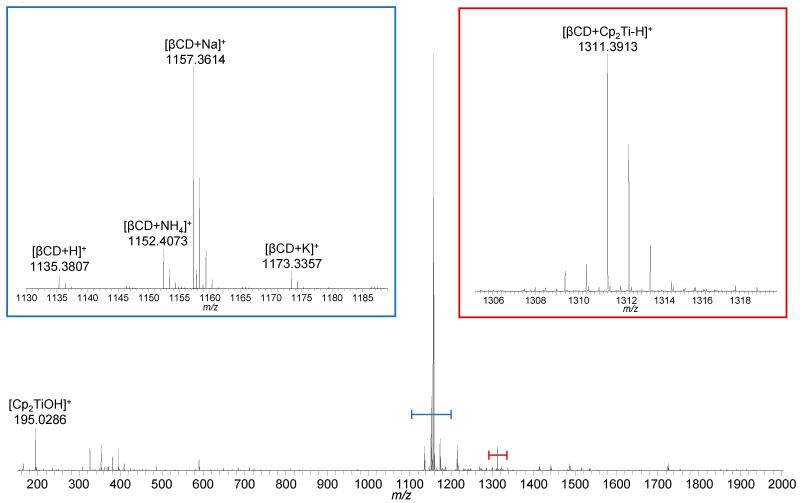
Full scan mass spectrum of the mixture of β-cyclodextrin and titanocene dichloride showing the peak indicating an interaction between the two compounds (red inset), as well as the hydrolyzed titanocene, and cationized β-cyclodextrin (blue).

Analysis of a 1:1 stoichiometric mixture of β-cyclodextrin and titanocene dichloride showed the ions arising from titanocene, mainly in hydrolyzed form as [Cp_2_Ti(OH)]^+^ (*m*/*z* 195.0286, 1.0 ppm), and from β-cyclodextrin (Figure 4, Supplementary materials Table S10). In addition, the isotopic distribution of the ion appearing at *m*/*z* 1311.3913 indicates an interaction between the titanium compound and β-cyclodextrin. Based on the accurate mass determination, the elemental composition of this ion was found to be C_52_H_79_O_35_Ti^+^, which corresponds to [βCD + Cp_2_Ti–H]^+^. Since the combination of β-cyclodextrin (C_42_H_70_O_35_) and Cp_2_Ti^2+^ (C_10_H_10_Ti^2+^) would result in a doubly charged complex with an elemental composition of C_52_H_80_O_35_Ti^2+^, one of the two charges must have been compensated for by the lack of a proton, presumably at a hydroxy group at either the primary or the secondary rim of β-cyclodextrin. More detailed localization of the deprotonation site, however, was not accomplishable by means of mass spectrometry.

**Figure 5:**
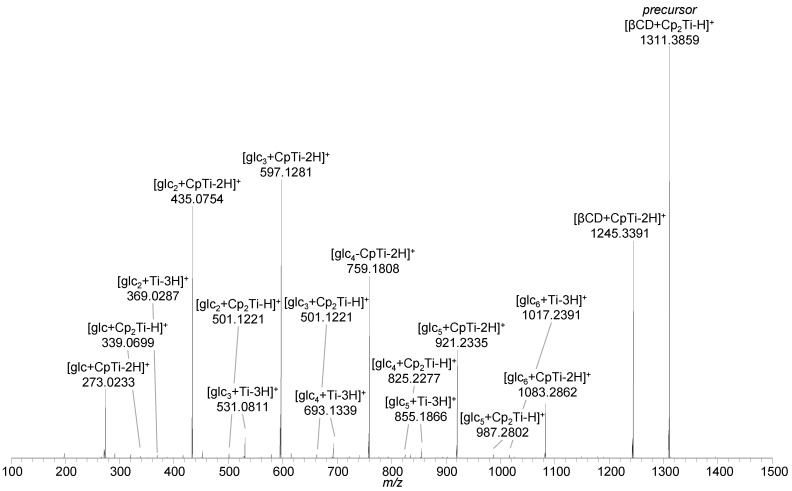
HCD mass spectrum of [βCD + Cp_2_Ti–H]^+^ (*m*/*z* 1311.40) at 47 eV (20% NCE) showing the loss of one or two cyclopentadiene moieties (C_5_H_6_) and the decomposition of β-cyclodextrin into its subunits, still interacting with titanocene species.

Collisional activation of [βCD + Cp_2_Ti–H]^+^ (*m*/*z* 1311.3859, −1.3 ppm) resulted in the loss of a neutral cyclopentadiene (C_5_H_6_) moiety, leading to [βCD + CpTi–2H]^+^ (*m*/*z* 1245.3391, −1.2 ppm). With increasing energy, the loss of neutral glucose subunits from β-cyclodextrin occurred, but the titanium species was still bound to the cyclodextrin fragment ions, as indicated by the [glc_n_ + Cp_2_Ti–H]^+^ (n = 1–6; *m*/*z* 339.0699, −2.1 ppm; *m*/*z* 501.1221, −2.8 ppm; *m*/*z* 663.1747, −2.4 ppm; *m*/*z* 825.2277, −1.7 ppm; *m*/*z* 987.2802, −1.7 ppm; *m*/*z* 1245.3391, −1.2 ppm) and [glc_n_ + CpTi–2H]^+^ (n = 1–6; *m*/*z* 273.0233, −1.5 ppm; *m*/*z* 435.0754, −2.5 ppm; *m*/*z* 597.1281, −2.0 ppm; *m*/*z* 759.1808, −1.8 ppm; *m*/*z* 921.2335, −1.6 ppm; *m*/*z* 1083.2862, −1.5 ppm) fragment ions. Loss of the second cyclopentadiene moiety in combination with the decomposition of β-cyclodextrin resulted in [glc_n_ + Ti–3H]^+^ (n = 2–6; *m*/*z* 369.0287, −2.4 ppm; *m*/*z* 531.0811, −2.4 ppm; *m*/*z* 693.1339, −1.9 ppm; *m*/*z* 855.1866, −1.6 ppm; *m*/*z* 1017.2391, −1.8 ppm) fragment ions (Figure 5, Supplementary materials Table S11). In contrast to β-cyclodextrin complexes with phenylalanine and oxaliplatin, separation of the host and guest structures was not observed as neither the intact β-cyclodextrin nor free titanocene-derived ions (e.g., [Cp_2_Ti(OH)]^+^) were detected and all fragment ions were found to contain titanium. These results indicate that the interaction of titanocene dichloride with cyclodextrin differs from the situation observed for the phenylalanine and oxaliplatin guest species.

Additional experiments probing the interaction of titanocene dichloride with the disaccharides sucrose and maltose gave evidence for the formation of singly charged [sugar + Cp_2_Ti–H]^+^ ions (*m*/*z* 519.1314, −5.1 ppm and *m*/*z* 519.1325, −3.1 ppm; Supplementary materials Tables S12 and S14), which is in agreement with the interaction observed for the titanocene with cyclodextrin. Collisional activation of this ion resulted in metallated fragment ions, such as [C_6_H_10_O_5_ + Cp_2_Ti–H]^+^ (*m*/*z* 339.0696, −3.2 ppm) and [C_6_H_12_O_6_ + Cp_2_Ti–H]^+^ (*m*/*z* 357.0800, −3.4 ppm) due to glycosidic bond cleavage and loss of one of the sugar moieties as a neutral (Supplementary materials Tables S13 and S15).

### 2.3. Methylated β-Cyclodextrins

The di- and trimethylated cyclodextrin analogs heptakis(2,6-di-*O*-methyl)-β-cyclodextrin (DMβ-cyclodextrin) and heptakis(2,3,6-tri-*O*-methyl)-β-cyclodextrin (TMβ-cyclodextrin) served as host structures for probing their interaction mode with titanocene and deciphering the role of the hydroxy groups. Full scan MS analyses of the host structures showed several peaks spaced by 14 mass units, indicating different degrees of methylation. Consequently, the spectral complexity was increased and the peak intensities were significantly reduced (Supplementary materials Tables S16 and S17) [42]. Nevertheless, the analysis of phenylalanine in the presence of the two methylated host structures revealed comparable interaction as with unmodified β-cyclodextrin, resulting in signals assigned as [DMβCD + Phe + H]^+^ (*m*/*z* 1496.6749, −0.1 ppm) and [TMβCD + Phe + H]^+^ (*m*/*z* 1594.7865, 1.1 ppm) (Supplementary materials Tables S18 and S22). The masking of the hydroxy groups at the rim of β-cyclodextrin does not prevent the interaction with phenylalanine and gives proof for the ability of methylated β-cyclodextrin to act as a host for hydrophobic structures.

**Figure 6:**
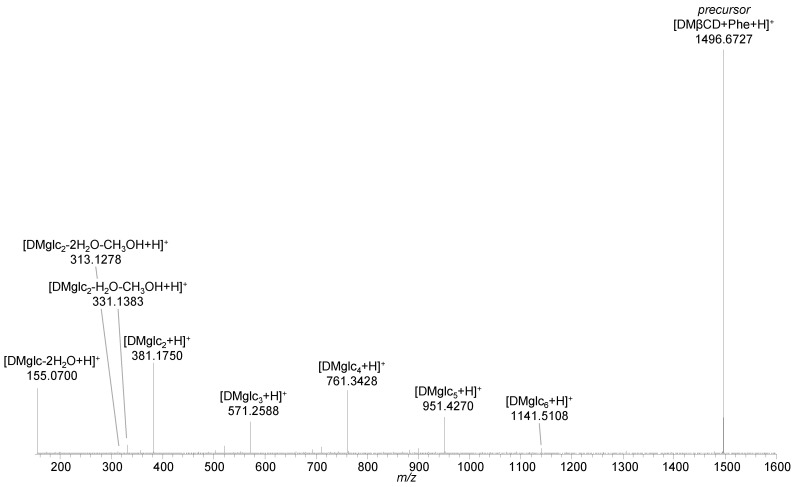
HCD mass spectrum of [DMβCD + Phe + H]^+^ (*m*/*z* 1496.70) at 40 eV (15% NCE) showing the separation of the precursor ion and subsequent decomposition of DMβ-cyclodextrin into its subunits [DMglc_n_ + H]^+^.

Collisional activation of the protonated [DMβCD + Phe + H]^+^ complexes led to fragmentation of the host structures, as demonstrated by Figure 6 for the complex with the dimethylated cyclodextrin (Supplementary materials Table S19). Though the product ion spectrum of [DMβCD + Phe + H]^+^ did not show the peaks of separated [Phe + H]^+^ and [DMβCD + H]^+^ ions, the lack of any signals referring to cyclodextrin fragments still bound to phenylalanine indicates a host-guest character of the interaction. A similar situation was encountered for trimethylated cyclodextrin as host for phenylalanine [TMβCD + Phe + H]^+^ (Supplementary materials Table S23).

The data obtained from mixtures of titanocene with methylated cyclodextrins significantly differ from previous results on unmodified cyclodextrin. As soon as all hydroxy groups at the rim of β-cyclodextrin are methylated, as in the case of trimethylated β-cyclodextrin, a peak referring to a [TMβCD + Cp_2_Ti–H]^+^ interaction product was not observed. This is in contrast to the experiments with unmodified β-cyclodextrin, which revealed the peak of [βCD + Cp_2_Ti–H]^+^. However, the peaks of the putative host-guest complexes [TMβCD + Cp_2_Ti(OH)_2_ + H]^+^ (*m*/*z* 1641.7401, 1.6 ppm) and [TMβCD + Cp_2_Ti(OH)]^+^ (*m*/*z* 1623.7297, 1.8 ppm) could be detected, though with minor abundance only, which hampered precursor ion isolation for tandem mass spectrometric analysis (Supplementary materials Table S24).

A different picture was seen for dimethylated and incompletely trimethylated cyclodextrins. The analysis of the mixture of titanocene dichloride with dimethylated β-cyclodextrin resulted in signals assigned as [DMβCD + Cp_2_Ti–H]^+^ (*m*/*z* 1507.6043; −1.6 ppm) as well as [DMβCD + Cp_2_Ti(OH)_2_ + H]^+^ (*m*/*z* 1543.6249, −1.9 ppm) (Supplementary materials Table S20). As previously observed for the β-cyclodextrin complex, the energy provided by collisional activation of [DMβCD + Cp_2_Ti–H]^+^ promotes the loss of a Cp-ligand (C_5_H_5_) yielding the [DMβCD + CpTi–H]^+^ (*m*/*z* 1442.5635, −2.8 ppm) fragment ion. Further fragment ions were found to correspond to titanium bound to glucose subunits, e.g., [DMglc_2_ + Cp_2_Ti–H]^+^ (*m*/*z* 557.1843, −3.2 ppm) (Supplementary materials Table S21).

The hydroxy groups occurring in incompletely trimethylated β-cyclodextrins (e.g., macrocycles bearing 19 (TMβCD*) or 20 (TMβCD^#^) instead of 21 methoxy groups only) represent targets for the interaction with titanocene. The peaks at *m*/*z* 1577.6880 and *m*/*z* 1591.7036, corresponding to [TMβCD* + Cp_2_Ti–H]^+^ (2.0 ppm) and [TMβCD^#^ + Cp_2_Ti–H]^+^ (1.9 ppm), respectively, clearly indicate that the lack of one or two methyl-groups is sufficient to form small amounts of covalently-bound titanocene adducts (Supplementary materials Table S23–S26).

### 2.4. Breakdown Curves

**Figure 7:**
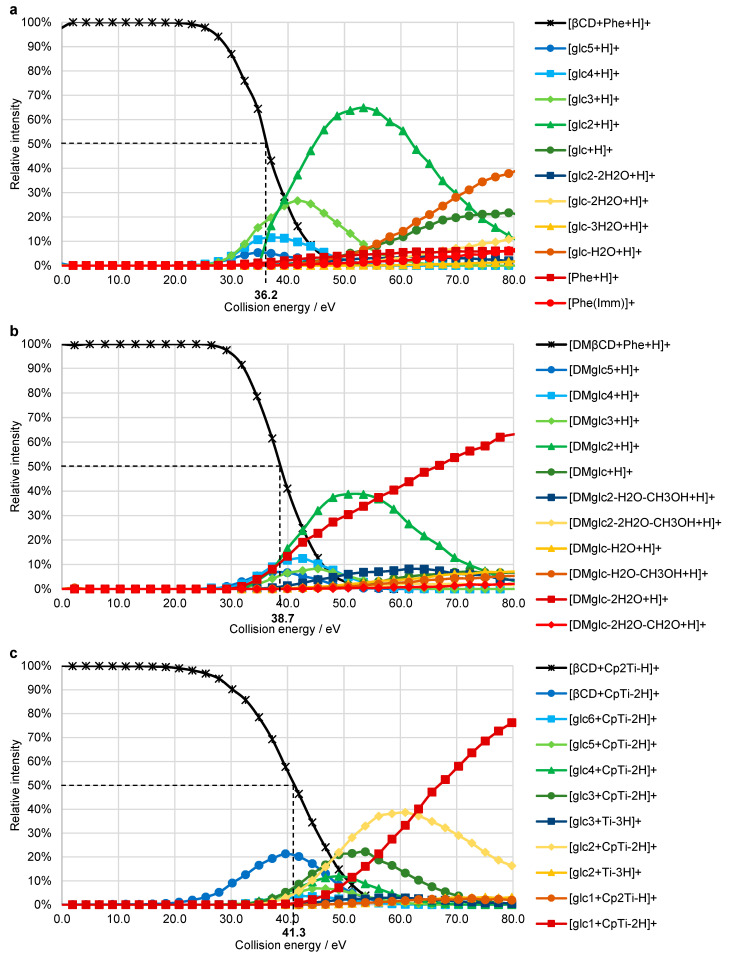
Breakdown curves recorded for HCD experiments of: (**a**) [βCD + Phe + H]^+^ (*m*/*z* 1300.48), (**b**) [DMβCD + Phe + H]^+^ (*m*/*z* 1496.70), (**c**) [βCD + Cp_2_Ti–H]^+^ (*m*/*z* 1311.40). Fragment ions with a maximum relative intensity <2% are not displayed. The dashed lines indicate the CE_50_ values.

To obtain a measure for the strength of the interaction between the host and guest molecules, breakdown curves were recorded by an incremental increase of the collision energy from 0% to 50% normalized collision energy (NCE) in HCD experiments. For each identified ion, the proportion of its intensity (*I_n_*) in the sum of the intensities of the precursor (*I_p_*) and all fragment ions (*I_f_*) is plotted against the collision energy ($y_{n}=I_{n}/\left(I_{p}+\sum I_{f}\right)\cdot 100$) [43]. CE_50_ values (50% of the precursor ion decomposed into fragments) of 36.2 eV (E_COM_ = 0.78 eV) and 38.7 eV (E_COM_ = 0.72 eV) were obtained for phenylalanine as guest in β-cyclodextrin, and for the interaction of phenylalanine with DMβ-cyclodextrin, respectively (Figure 7a,b, Supplementary materials Figures S1 and S2). The CE_50_ value of 34.9 eV (E_COM_ = 0.64 eV), determined for the decomposition of [βCD + oxaliPt + H]^+^, is on the same scale, thus, strengthening the hypothesis of host-guest complex formation (Supplementary materials Figure S4).

The breakdown curve of [βCD + Cp_2_Ti–H]^+^ shows a CE_50_ value of 41.3 eV (E_COM_ = 0.88 eV, Figure 7c, Supplementary materials Figure S3), which is significantly higher than for the host-guest complexes formed between phenylalanine and DMβ- or β-cyclodextrin. Due to the loss of the first Cp-ligand occurring at low collision energies prior to the decomposition of the cyclodextrin, the slope of the precursor decomposition curve is rather flat.

## 3. Discussion

Experiments with phenylalanine and oxaliplatin demonstrated the ability of electrospray ionization mass spectrometry to detect host-guest complexes. Though the literature suggests the formation of an inclusion complex of titanocene dichloride with β-cyclodextrin [19,38], mass spectrometric experiments did not give evidence for the generation of the corresponding [βCD + Cp_2_TiCl_2_ + H]^+^ ion at *m*/*z* 1383.3409. Titanocene dichloride is known to rapidly undergo hydrolysis of the chloride ligands upon contact with water. Nevertheless, the inclusion of these hydrolysis products in β-cyclodextrin was not detected, but the corresponding peaks were observed with low abundance in the spectra with DMβ- and TMβ-cyclodextrin hosts. On the other hand, experiments with β-cyclodextrins bearing unmodified hydroxy groups and disaccharides resulted in similar ions of the type [sugar + Cp_2_Ti–H]^+^, clearly indicating the significance of hydroxy groups on the interaction of titanocene with saccharides.

**Figure 8:**
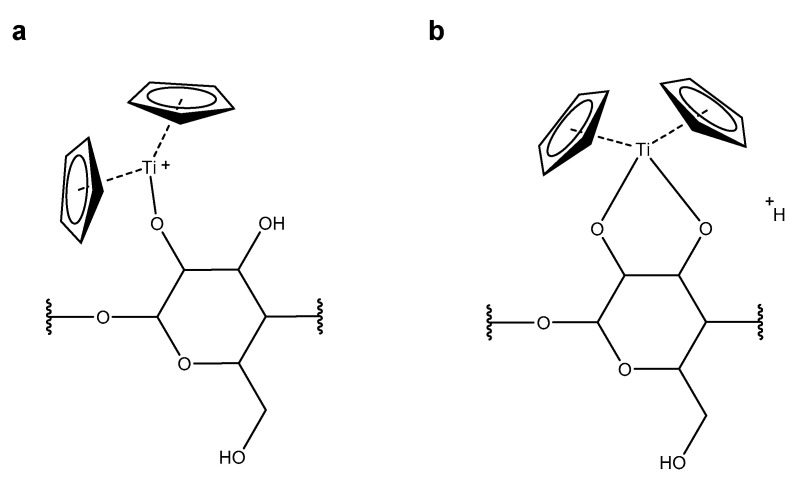
Possible interaction modes between titanocene and β-cyclodextrin: (**a**) formation of one covalent bond and the positive charge residing on the titanium; (**b**) formation of two covalent bonds with the charge emerging from an additional proton.

Based on the elemental composition determined for the titanocene-cyclodextrin assembly [βCD + Cp_2_Ti–H]^+^, two interaction modes are possible: (i) formation of a single covalent bond between the titanium center and a hydroxy group of the primary or secondary rim of β-cyclodextrin with the charge residing on the titanium (Figure 8a), and (ii) formation of two covalent bonds to hydroxy groups while the charge is provided by an additional proton (Figure 8b). Elucidation of the elemental composition does not provide any further details about the mode of interaction, nor the position and number of the hydroxy groups involved. Electrospray mass spectrometric analysis of cyclodextrin, even in a mixture with the metallocene, resulted in the signal of the protonated macrocycle (*m*/*z* 1135.3807, 3.3 ppm) and additionally, in the highly abundant peak of the cyclodextrin-sodium adduct (*m*/*z* 1157.3614, 2.1 ppm). Sodium adduct formation ([βCD + Cp_2_Ti–2H + Na]^+^, *m*/*z* 1333.3695), however, was never observed for the titanocene-cyclodextrin assembly. In the case of interaction mode ii, this peak is anticipated, as any cation might provide the charge for the assembly. The lack of this ion suggests the formation of a single covalent bond between any deprotonated hydroxy group of β-cyclodextrin and the titanium coordination center as the most plausible mode of interaction, where the excess positive charge resides on the titanium (Figure 8a). This finding is supported by the results from collisional activation experiments of [βCD + Cp_2_Ti–H]^+^, which gave evidence for cyclodextrin fragments still interacting with titanium. According to our previous work [42], bond cleavage between the glucopyranose units as the predominant mechanism for the decomposition of β-cyclodextrin is independent of the hydroxy groups and, therefore, not affected by the interaction with titanocene.

The CE_50_ values determined for [βCD + Phe + H]^+^, [DMβCD + Phe + H]^+^, and [βCD + Cp_2_Ti–H]^+^ are significantly higher than the 15.3 eV obtained for [βCD + H]^+^ in a previous study [42]. Likewise, Dossmann et al. [44] reported values ranging between 15 and 23 eV for doubly charged metallated β-cyclodextrin ions [βCD + M]^2+^ (M = Fe^2+^, Co^2+^, Ni^2+^, Cu^2+^, Zn^2+^). At first sight, these results are unexpected as the dissociation of a covalent bond requires more energy than the disruption of a non-covalent interaction. However, a proton or metal ion bound to glycosidic oxygen significantly destabilizes the corresponding glycosidic bond, thereby inducing dissociation. Protonation of the phenylalanine inclusion complex, on the other hand, occurs most likely at the N-terminal amino group of the amino acid, therefore not affecting the integrity of the host structure, which is reflected by the higher CE_50_ value. 

The hypothesis of the formation of titanocene dichloride inclusion complexes with β-cyclodextrin, as previously proposed from NMR experiments [38] and theoretical approaches [19], could not be confirmed by our mass spectrometric results, as the formation of a host-guest complex without the involvement of covalent bonds would result in disintegration of the complex, as shown for the interaction of β-cyclodextrin with phenylalanine and oxaliplatin. Therefore, unmodified cyclodextrins are not expected to considerably improve the hydrolytic stability and bioavailability of titanocene dichloride.

## 4. Materials and Methods

D-L-Phenylalanine (Sigma Aldrich, Buchs, Switzerland) was dissolved in 50/50 water/acetonitrile + 0.5% formic acid (water: MilliQ, in house; acetonitrile: Biosolve, Valkenswaard, the Netherlands; formic acid: Sigma Aldrich, Buchs, Switzerland) to give a 50 mM solution and further diluted to 1 mM in 50/50 water/acetonitrile. Oxaliplatin and titanocene dichloride (Sigma Aldrich, Buchs, Switzerland) were dissolved in ULC-MS grade acetonitrile (Biosolve, Valkenswaard, the Netherlands) and ultrasonicated for 30 min to give a 1 mM stock solution. Heptakis(2,3,6-tri-*O*-methyl)-β-cyclodextrin (TMβ-cyclodextrin), heptakis(2,6-di-*O*-methyl)-β-cyclodextrin (DMβ-cylclodextrin) (Sigma Aldrich, Buchs, Switzerland), β-cyclodextrin (Fluka, Sigma Aldrich, Buchs, Switzerland), sucrose (Merck, Darmstadt, Germany), and maltose (Sigma Aldrich, Buchs, Switzerland) were dissolved in MilliQ water to a final concentration of 1 mM by shaking for 1 h at 21 °C and 12,000 rpm on a thermomixer (Eppendorf, Schönenbuch, Switzerland).

For measurement, samples at a concentration of 0.02 mM were prepared either in 50/50 water/acetonitrile, 50/50 water/acetonitrile + 0.5% formic acid, or methanol (Sigma Aldrich, Bruchs, Switzerland), either of the host and guest structures solely, or mixed in a 1:1 molar ratio.

### Mass Spectrometry

Nano electrospray-ionization mass spectrometric experiments were performed on an LTQ Orbitrap XL instrument (Thermo Fisher Scientific, Bremen, Germany) with Econo12 Glass PicoTips (New Objective, Littleton, MA, USA) as electrospray emitters. Mass spectrometry was performed in positive ionization mode with spray voltages ranging from 0.8 to 1.3 kV, tube lens voltage of 250 V, capillary voltage of 20 V, and a capillary temperature of 200 °C. The mass spectrometer was used in FTMS mode at a resolution of 100,000. HCD experiments were performed with precursor ion isolation windows in the range of 3 to 8 *m*/*z*. Collision energies ranged between 0% and 50% NCE and were converted to eV [45,46]. Data processing was performed using the Xcalibur software suite (Thermo Fisher Scientific, Bremen, Germany).

## Data Availability

Data are contained within the Supplementary Materials.

## Supplementary Materials

Supplementary Materials can be found at https://www.mdpi.com/article/10.3390/ijms22189789/s1.
