# Supplementary material for: Mass Spectrometric Evaluation of β-Cyclodextrins as Potential Hosts for Titanocene Dichloride
Source: Int J Mol Sci. 2021 Sep 10;22(18):9789. doi: 10.3390/ijms22189789 (PMC8467183; doi:10.3390/ijms22189789)
Supplement: Supplementary file 1 [file ijms-22-09789-s001.zip › ijms-1347633-supplementary.pdf]

Supplementary Materials

Mass spectrometric investigation of β-cyclodextrins as hosts for titanocene dichloride

Pia S. Bruni [1] and Stefan Schürch [1,*]

[1] Department of Chemistry, Biochemistry and Pharmaceutical Sciences, University of Bern, 3012 Bern, Switzerland; pia.bruni@unibe.ch

* Correspondence: stefan.schuerch@unibe.ch

Table S1. Full scan mass spectrum of β-cyclodextrin in 50/50 H_2O/MeCN (100% = 6.19E7).
Table S2. Full scan mass spectrum of phenylalanine in 50/50 H_2O/MeCN (100% = 9.45E7).
Table S3. Full scan mass spectrum of oxaliplatin in 50/50 H_2O/MeCN (100% = 5.01E6).
Table S4. Full scan mass spectrum of titanocene dichloride (Cp_2TiCl_2) in 50/50 H_2O/MeCN +1% FA (100% = 2.33E7).
Table S5. Full scan mass spectrum of titanocene dichloride (Cp_2TiCl_2) in MeOH (100% = 7.30E5).
Table S6. Full scan mass spectrum of the β-cyclodextrin/phenylalanine mixture in 50/50 H_2O/MeCN (100% = 2.75E7).
Table S7. Tandem mass spectrum of the β-cyclodextrin/phenylalanine mixture in 50/50 H_2O/MeCN. Precursor ion: *m/z* 1300.48, HCD at 16% NCE (37 eV), 100% = 9.23E5.
Table S8. Full scan mass spectrum of the β-cyclodextrin/oxaliplatin mixture in 50/50 H_2O/MeCN (100% = 7.97E6).
Table S9. Tandem mass spectrum of the β-cyclodextrin/oxaliplatin mixture in 50/50 H_2O/MeCN. Precursor ion: *m/z* 1532.50, HCD at 14% NCE (38 eV), 100% = 1.64E5.
Table S10. Full scan mass spectrum of the β-cyclodextrin/titanocene dichloride mixture in 50/50 H_2O/MeCN (100% = 2.19E7).
Table S11. Tandem mass spectrum of the β-cyclodextrin/titanocene dichloride mixture in 50/50 H_2O/MeCN. Precursor ion: $[βCD+Cp_2Ti-H]^+$ *m/z* 1311.40, HCD at 20% NCE (47 eV), 100% = 8.82E5.
Table S12. Full scan mass spectrum of the sucrose/titanocene dichloride mixture in 50/50 H_2O/MeCN (100% = 3.67E7).
Table S13. Tandem mass spectrum of the sucrose/titanocene dichloride mixture in 50/50 H_2O/MeCN. Precursor ion: $[suc+Cp_2Ti-H]^+$ *m/z* 519.20, HCD at 19% NCE (17 eV), 100% = 2.17E5.
Table S14. Full scan mass spectrum of the maltose/titanocene dichloride mixture in 50/50 H_2O/MeCN (100% = 1.06E7).
Table S15. Tandem mass spectrum of the maltose/titanocene dichloride mixture in 50/50 H_2O/MeCN. Precursor ion: $[maltose+Cp_2Ti-H]^+$ *m/z* 519.20, HCD at 20% NCE (18 eV), 100% = 2.20E5.
Table S16. Full scan mass spectrum of DMβ-cyclodextrin in 50/50 H_2O/MeCN +1% FA (100% = 2.94E7).
Table S17. Full scan mass spectrum of TMβ-cyclodextrin in 50/50 H_2O/MeCN (100% = 4.86E6).
Table S18. Full scan mass spectrum of the DMβ-cyclodextrin/phenylalanine mixture in 50/50 H_2O/MeCN (100% = 1.08E7).
Table S19. Tandem mass spectrum of the DMβ-cyclodextrin/phenylalanine mixture in 50/50 H_2O/MeCN. Precursor ion: $[DMβCD+Phe+H]^+$ *m/z* 1496.70, HCD at 15% NCE (40 eV), 100% = 1.08E6.
Table S20. Full scan mass spectrum of the DMβ-cyclodextrin/titanocene dichloride mixture in 50/50 H_2O/MeCN (100% = 7.57E7).
Table S21. Tandem mass spectrum of the DMβ-cyclodextrin/titanocene dichloride mixture in 50/50 H_2O/MeCN. Precursor ion: $[DMβCD+Cp_2Ti-H]^+$ *m/z* 1507.60, HCD at 20% NCE (54 eV), 100% = 1.18E6.
Table S22. Full scan mass spectrum of the TMβ-cyclodextrin/phenylalanine mixture in 50/50 H_2O/MeCN (100% = 7.74E7).
Table S23. Tandem mass spectrum of the TMβ-cyclodextrin/phenylalanine mixture in 50/50 H_2O/MeCN. Precursor ion: $[TMβCD+Phe+H]^+$ *m/z* 1594.80, HCD at 15% NCE (43 eV), 100% = 2.22E5.
Table S24. Full scan mass spectrum of the TMβ-cyclodextrin/titanocene dichloride mixture in 50/50 H2O/MeCN (100% = 4.65E7).
Table S25. Tandem mass spectrum of the TMβ-cyclodextrin/phenylalanine mixture in 50/50 H_2O/MeCN + 1% FA. Precursor ion: $[TMβCD^*+Phe+H]^+$ with a total of 19 methyl groups at *m/z* 1566.80, HCD at 15% NCE (42 eV), 100% = 3.53E5.
Table S26. Tandem mass spectrum of the TMβ-cyclodextrin/phenylalanine mixture in 50/50 H_2O/MeCN + 1% FA. Precursor ion: $[TMβCD^{\#}+Phe+H]^+$ with a total of 20 methyl groups at *m/z* 1580.80, HCD at 15% NCE (42 eV), 100% = 6.11E5.
Figure S1. Tandem mass spectra of the β-cyclodextrin/phenylalanine mixture underlying the breakdown curve of $[βCD+Phe+H]^+$.
Figure S2. Tandem mass spectra of the DMβ-cyclodextrin/phenylalanine mixture underlying the breakdown curve of $[DMβCD+Phe+H]^+$.
Figure S3. Tandem mass spectra of the β-cyclodextrin/titanocene dichloride mixture underlying the breakdown curve of $[βCD+Cp_2Ti-H]^+$.
Figure S4. Tandem mass spectra of the β-cyclodextrin/oxaliplatin mixture underlying the breakdown curve of $[βCD+oxaliPt+H]^+$.

Table S1. Full scan mass spectrum of β-cyclodextrin in 50/50 H_2O/MeCN (100% = 6.19E7).

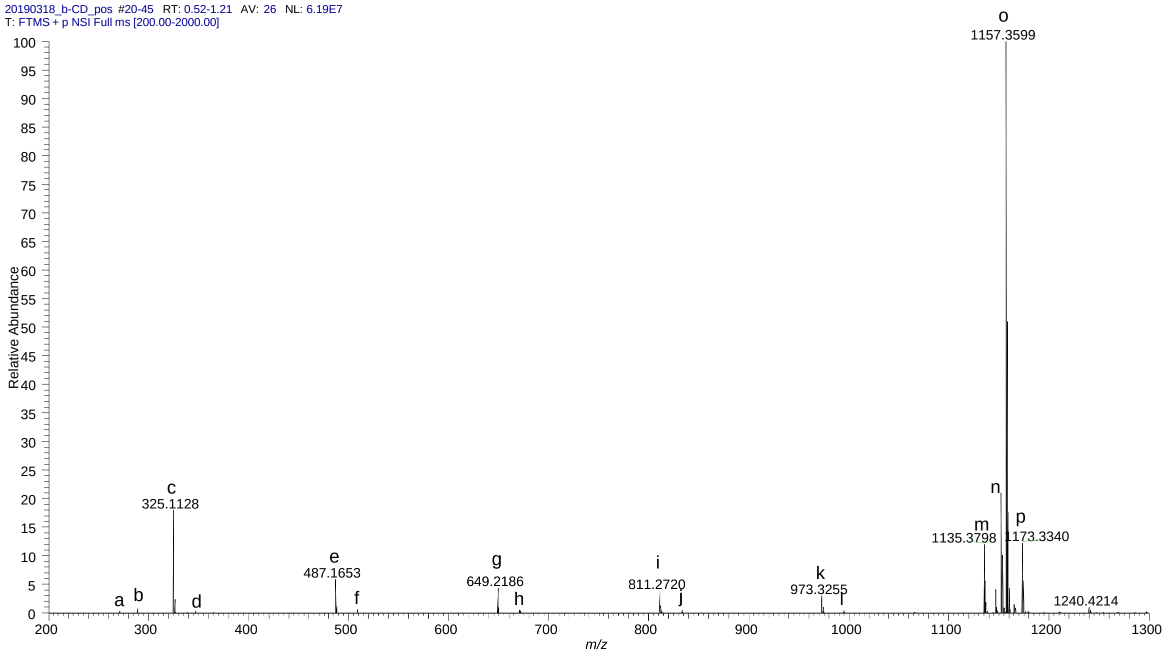

	m/z experimental	Name	*m/z* calculation	Error
a	271.0812	$[glc_2\text{-}3H_2O+H]^+$	271.0812	0.0 ppm
b	289.0917	$[glc_2\text{-}2H_2O+H]^+$	289.0918	-0.3 ppm
c	325.1128	$[glc_2+H]^+$	325.1129	-0.3 ppm
d	347.0947	$[glc_2+Na]^+$	347.0949	-0.6 ppm
e	487.1653	$[glc_3+H]^+$	487.1657	-0.8 ppm
f	509.1471	$[glc_3+Na]^+$	509.1477	-1.2 ppm
g	649.2186	$[glc_4+H]^+$	649.2186	0.0 ppm
h	671.2004	$[glc_4+Na]^+$	671.2005	-0.1 ppm
i	811.2720	$[glc_5+H]^+$	811.2714	0.7 ppm
j	833.2538	$[glc_5+Na]^+$	833.2533	0.6 ppm
k	973.3255	$[glc_6+H]^+$	973.3242	1.3 ppm
l	995.3073	$[glc_6+Na]^+$	995.3062	1.1 ppm
m	1135.3798	$[\beta CD+H]^+$	1135.3770	2.5 ppm
n	1152.4060	$[\beta CD+NH_4]^+$	1152.4036	2.1 ppm
o	1157.3599	$[\beta CD+Na]^+$	1157.3590	0.8 ppm
p	1173.3340	$[\beta CD+K]^+$	1173.3329	0.9 ppm

Table S2. Full scan mass spectrum of phenylalanine in 50/50 H_2O/MeCN (100% = 9.45E7).

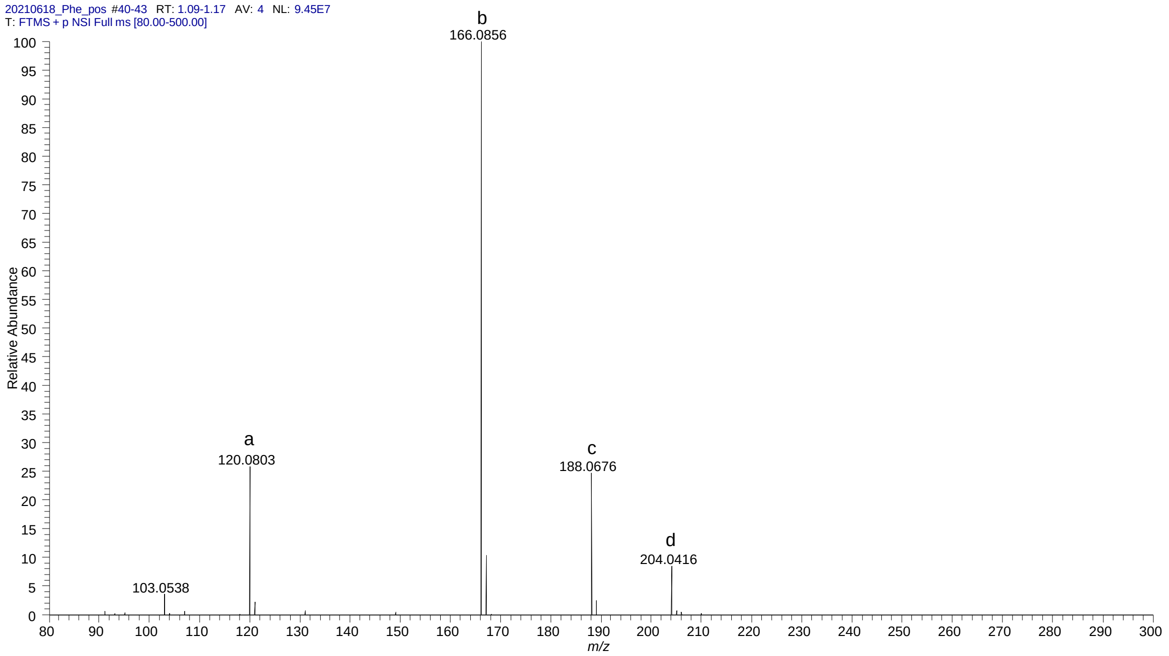

	m/z experimental	Name	*m/z* calculation	Error
a	120.0803	$[Imm(Phe)]^+$	120.0808	-4.2 ppm
b	166.0856	$[Phe+H]^+$	166.0863	-4.2 ppm
c	188.0676	$[Phe+Na]^+$	188.0682	-3.2 ppm
d	204.0416	$[Phe+K]^+$	204.0421	-2.5 ppm

Table S3. Full scan mass spectrum of oxaliplatin in 50/50 H_2O/MeCN (100% = 5.01E6).

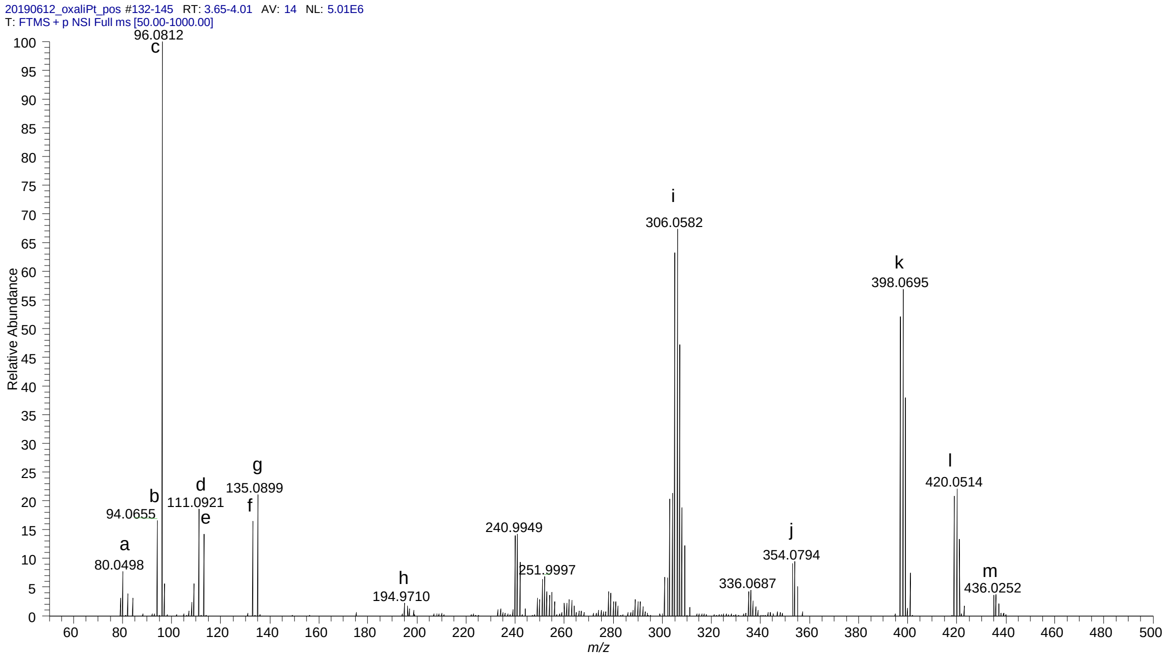

	m/z experimental	Name	*m/z* calculation	Error
a	80.0498	$[C_5H_6N]^+$	80.0495	3.7 ppm
b	94.0655	$[C_6H_6N]^+$	94.0655	0.0 ppm
c	96.0812	$[C_6H_{10}N]^+$	96.0808	4.2 ppm
d	111.0921	$[C_6H_{11}N_2]^+$	111.0917	3.6 ppm
e	113.1078	$[C_6H_{13}N_2]^+$	113.1073	4.4 ppm
f	133.0742	$[C_6H_{10}N_2Na]^+$	133.0736	4.5 ppm
g	135.0899	$[C_6H_{12}N_2Na]^+$	135.0893	4.4 ppm
h	194.9653	$[Pt]^+$	194.9642	5.6 ppm
i	306.0582	$[oxaliPt\text{-}C_2H_4O_4\text{+}H]^+$	306.0564	5.9 ppm
j	354.0794	$[oxaliPt\text{-}CO_2\text{+}H]^+$	354.0776	5.1 ppm
k	398.0695	$[oxaliPt\text{+}H]^+$	398.0674	5.3 ppm
l	420.0514	$[oxaliPt\text{+}Na]^+$	420.0494	4.8 ppm
m	436.0252	$[oxaliPt\text{+}K]^+$	436.0233	4.4 ppm

Table S4. Full scan mass spectrum of titanocene dichloride (Cp_2TiCl_2) in 50/50 H_2O/MeCN +1% FA (100% = 2.33E7).

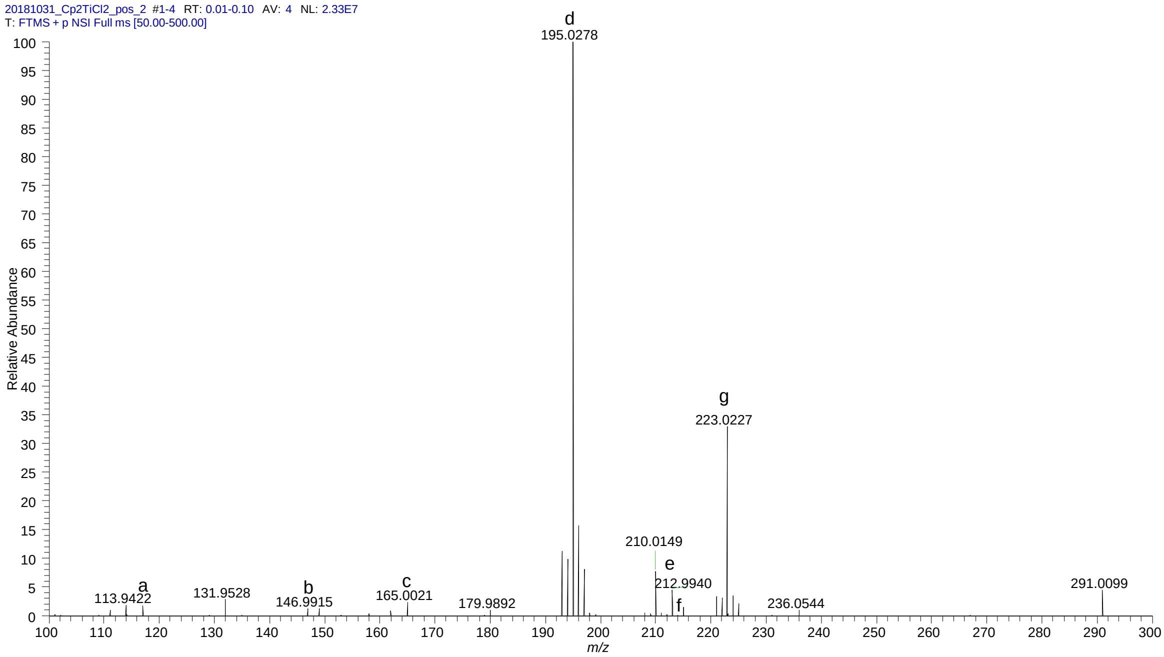

	m/z experimental	Name	*m/z* calculation	Error
a	116.9657	$[Ti(OH)_3{+}(H_2O)]^+$	116.9662	-4.3 ppm
b	146.9915	$[CpTi(OH)_2]^+$	146.9920	-3.4 ppm
c	165.0021	$[CpTi(OH)_2{+}(H_2O)]^+$	165.0026	-3.0 ppm
d	195.0278	$[Cp_2Ti(OH)]^+$	195.0284	-3.1 ppm
e	212.9940	$[Cp_2TiCl]^+$	212.9945	-2.3 ppm
f	213.0384	$[Cp_2Ti(OH){+}(H_2O)]^+$	213.0390	-2.7 ppm
g	223.0227	$[Cp_2Ti(COOH)]^+$	223.0233	-2.7 ppm

Table S5. Full scan mass spectrum of titanocene dichloride (Cp_2TiCl_2) in MeOH (100% = 7.30E5).

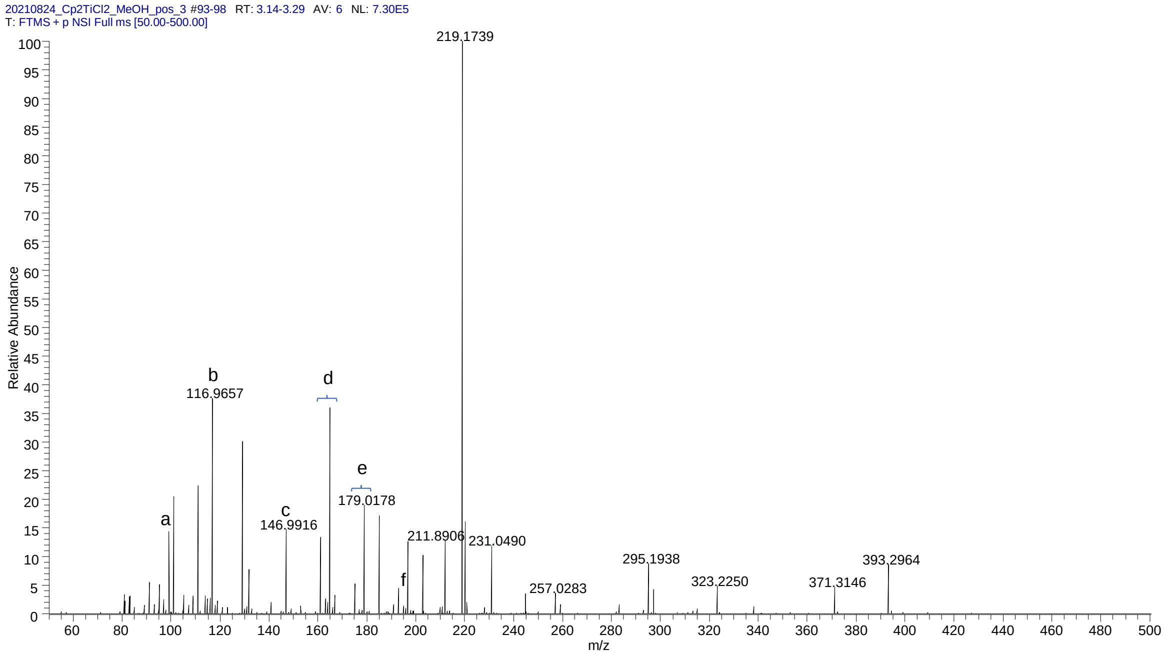

	m/z experimental	Name	*m/z* calculation	Error
a	98.9552	$[Ti(OH)_3]^+$	98.9556	-4.0 ppm
b	116.9657	$[Ti(OH)_3+(H_2O)]^+$	116.9662	-4.3 ppm
c	146.9916	$[CpTi(OH)_2]^+$	146.9920	-2.7 ppm
d	161.0073	$[CpTi(OH)(OCH_3)]^+$	161.0077	-2.5 ppm
	165.0022	$[CpTi(OH)_2+(H_2O)]^+$	165.0026	-2.4 ppm
e	175.0229	$[CpTi(OCH_3)_2]^+$	175.0233	-2.3 ppm
	179.0178	$[CpTi(OH)(OCH_3)(H_2O)]^+$	179.0182	-2.2 ppm
f	193.0334	$[CpTi(OH)(OCH_3)_2+H]^+$	193.0339	-2.6 ppm

Table S6. Full scan mass spectrum of the β-cyclodextrin/phenylalanine mixture in 50/50 H_2O/MeCN (100% = 2.75E7).

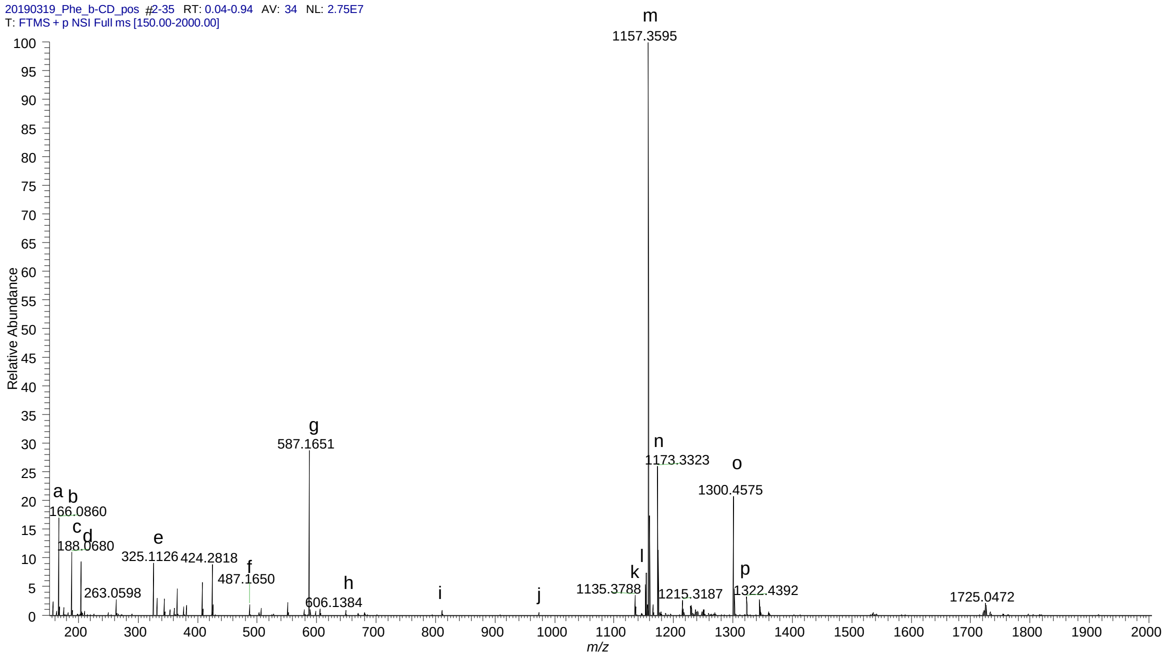

	m/z experimental	Name	*m/z* calculation	Error
a	163.0599	$[glc_1+H]^+$	163.0601	-1.2 ppm
b	166.0860	$[Phe+H]^+$	166.0863	-1.8 ppm
c	188.0680	$[Phe+Na]^+$	188.0682	-1.1 ppm
d	204.0420	$[Phe+K]^+$	204.0421	-0.5 ppm
e	325.1126	$[glc_2+H]^+$	325.1129	-0.9 ppm
f	487.1650	$[glc_3+H]^+$	487.1657	-1.4 ppm
g	587.1651	$[\beta CD+Ca]^{2+}$	587.1656	-1.0 ppm
h	649.2180	$[glc_4+H]^+$	649.2186	-0.9 ppm
i	811.2713	$[glc_5+H]^+$	811.2714	-0.1 ppm
j	973.3247	$[glc_6+H]^+$	973.3242	0.5 ppm
k	1135.3788	$[\beta CD+H]^+$	1135.3770	1.6 ppm
l	1152.4054	$[\beta CD+NH_4]^+$	1152.4036	1.6 ppm
m	1157.3595	$[\beta CD+Na]^+$	1157.3590	0.4 ppm
n	1173.3323	$[\beta CD+K]^+$	1173.3329	-0.5 ppm
o	1300.4575	$[\beta CD+Phe+H]^+$	1300.4560	1.2 ppm
p	1322.4392	$[\beta CD+Phe+Na]^+$	1322.4380	0.9 ppm

Table S7. Tandem mass spectrum of the β-cyclodextrin/phenylalanine mixture in 50/50 H_2O/MeCN. Precursor ion: *m/z* 1300.48, HCD at 16% NCE (37 eV), 100% = 9.23E5.

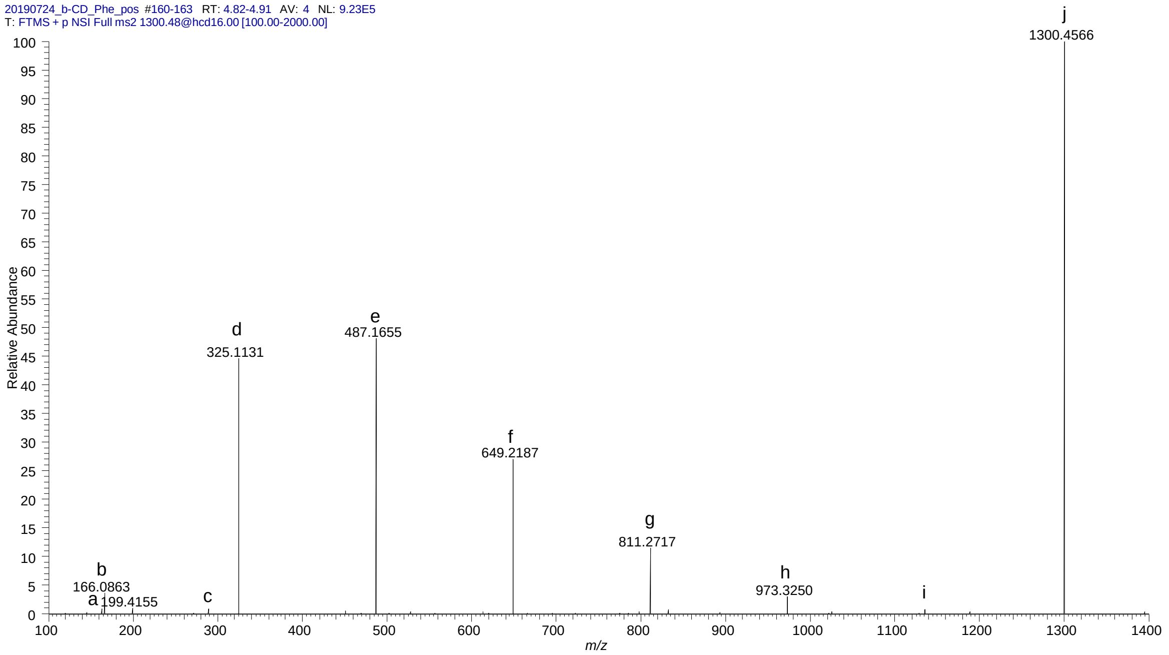

	m/z experimental	Name	*m/z* calculation	Error
a	163.0601	$[glc+H]^+$	163.0601	0.0 ppm
b	166.0863	$[Phe+H]^+$	166.0863	0.0 ppm
c	289.0920	$[glc_2-2H_2O+H]^+$	289.0918	0.7 ppm
d	325.1131	$[glc_2+H]^+$	325.1129	0.6 ppm
e	487.1655	$[glc_3+H]^+$	487.1657	-0.4 ppm
f	649.2187	$[glc_4+H]^+$	649.2186	0.2 ppm
g	811.2717	$[glc_5+H]^+$	811.2714	0.4 ppm
h	973.3250	$[glc_6+H]^+$	973.3242	0.8 ppm
i	1135.3777	$[\beta CD+H]^+$	1135.3770	0.6 ppm
j	1300.4566	$[\beta CD+Phe+H]^+$	1300.4560	0.5 ppm

Table S8. Full scan mass spectrum of the β-cyclodextrin/oxaliplatin mixture in 50/50 H_2O/MeCN (100% = 7.97E6).

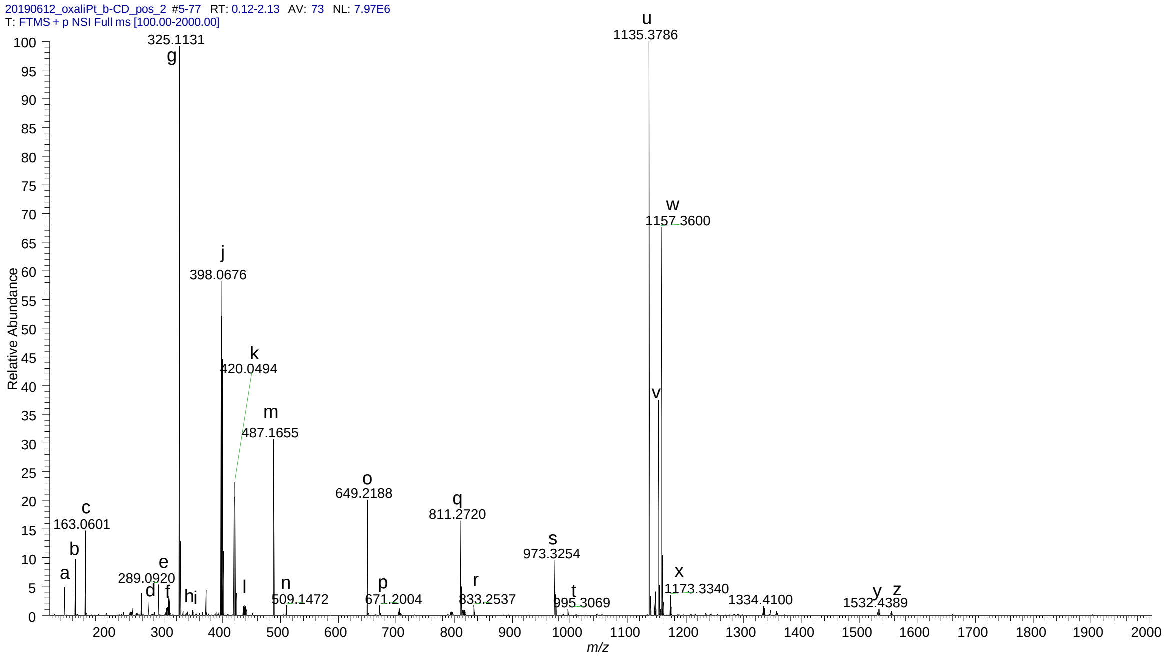

	m/z experimental	Name	*m/z* calculation	Error
a	127.0389	$[glc\text{-}2H_2O+H]^+$	127.0390	-0.8 ppm
b	145.0495	$[glc\text{-}H_2O+H]^+$	145.0495	0 .0 ppm
c	163.0601	$[glc+H]^+$	163.0601	0.0 ppm
d	271.0814	$[glc_2\text{-}3H_2O+H]^+$	271.0812	0.7 ppm
e	289.0920	$[glc_2\text{-}2H_2O+H]^+$	289.0918	0.7 ppm
f	306.0568	$[oxaliPt\text{-}C_2H_4O_4+H]^+$	306.0564	1.3 ppm
g	325.1131	$[glc_2+H]^+$	325.1129	0.6 ppm
h	347.0948	$[glc_2+Na]^+$	347.0949	-0.3 ppm
i	354.0777	$[oxaliPt\text{-}CO_2+H]^+$	354.0776	0.3 ppm
j	398.0676	$[oxaliPt+H]^+$	398.0674	0.5 ppm
k	420.0494	$[oxaliPt+Na]^+$	420.0494	0.0 ppm
l	436.0232	$[oxaliPt+K]^+$	436.0233	-0.2 ppm
m	487.1655	$[glc_3+H]^+$	487.1657	-0.4 ppm
n	509.1472	$[glc_3+Na]^+$	509.1477	-1.0 ppm
o	649.2188	$[glc_4+H]^+$	649.2186	0.3 ppm
p	671.2004	$[glc_4+Na]^+$	671.2005	-0.1 ppm
q	811.2720	$[glc_5+H]^+$	811.2714	0.7 ppm
r	833.2537	$[glc_5+Na]^+$	833.2533	0.5 ppm
s	973.3254	$[glc_6+H]^+$	973.3242	1.2 ppm
t	995.3069	$[glc_6+Na]^+$	995.3062	0.7 ppm
u	1135.3786	$[\beta CD+H]^+$	1135.3770	1.4 ppm
v	1152.4052	$[\beta CD+NH_4]^+$	1152.4036	1.4 ppm
w	1157.3600	$[\beta CD+Na]^+$	1157.3590	0.9 ppm
x	1173.3340	$[\beta CD+K]^+$	1173.3329	0.9 ppm
y	1532.4389	$[\beta CD+oxaliPt+H]^+$	1532.4372	1.1 ppm
z	1554.4207	$[\beta CD+oxaliPt+Na]^+$	1554.4191	1.0 ppm

Table S9. Tandem mass spectrum of the β-cyclodextrin/oxaliplatin mixture in 50/50 H_2O/MeCN. Precursor ion: *m*/*z* 1532.50, HCD at 14% NCE (38 eV), 100% = 1.64E5.

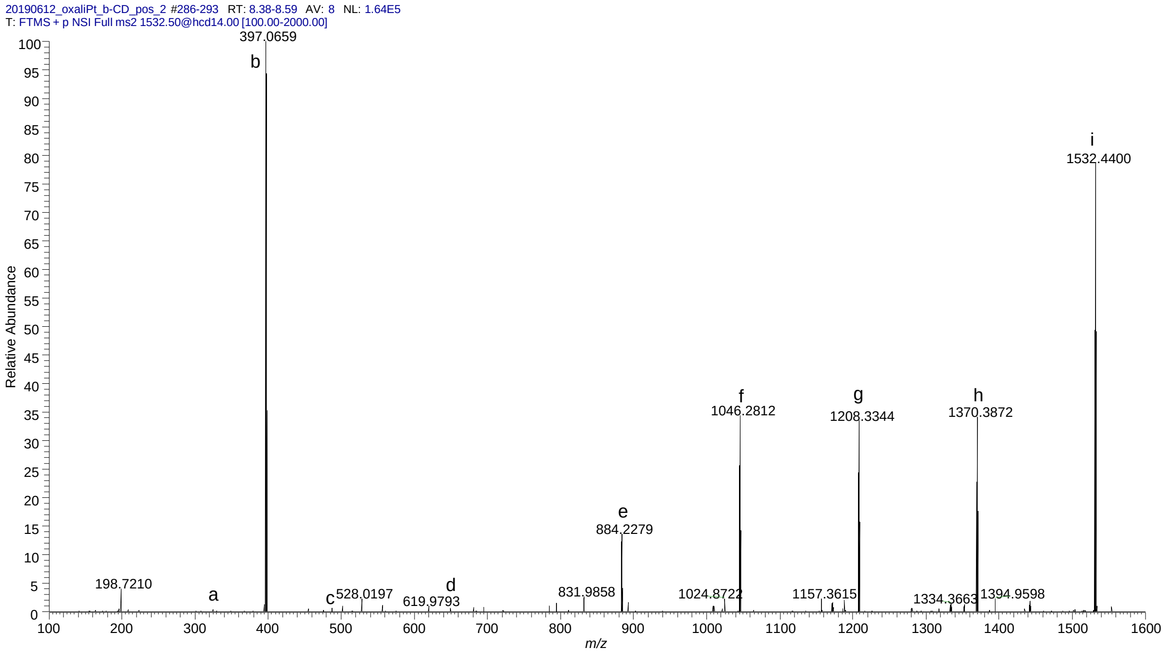

	m/*z* experimental	Name	*m*/*z* calculation	Error
a	325.1134	$[glc_2+H]^+$	325.1129	1.5 ppm
b	398.0681	$[oxaliPt+H]^+$	398.0674	1.8 ppm
c	487.1661	$[glc_3+H]^+$	487.1657	0.8 ppm
d	649.2195	$[glc_4+H]^+$	649.2186	1.4 ppm
e	884.2279	$[glc_3+oxaliPt+H]^+$	884.2259	2.3 ppm
f	1046.2812	$[glc_4+oxaliPt+H]^+$	1046.2787	2.4 ppm
g	1208.3344	$[glc_5+oxaliPt+H]^+$	1208.3315	2.4 ppm
h	1370.3872	$[glc_6+oxaliPt+H]^+$	1370.3843	2.1 ppm
i	1532.4400	[βCD+oxaliPt+H]$^+$	1532.4372	1.8 ppm

Table S10. Full scan mass spectrum of the β-cyclodextrin/titanocene dichloride mixture in 50/50 H_2O/MeCN (100% = 2.19E7).

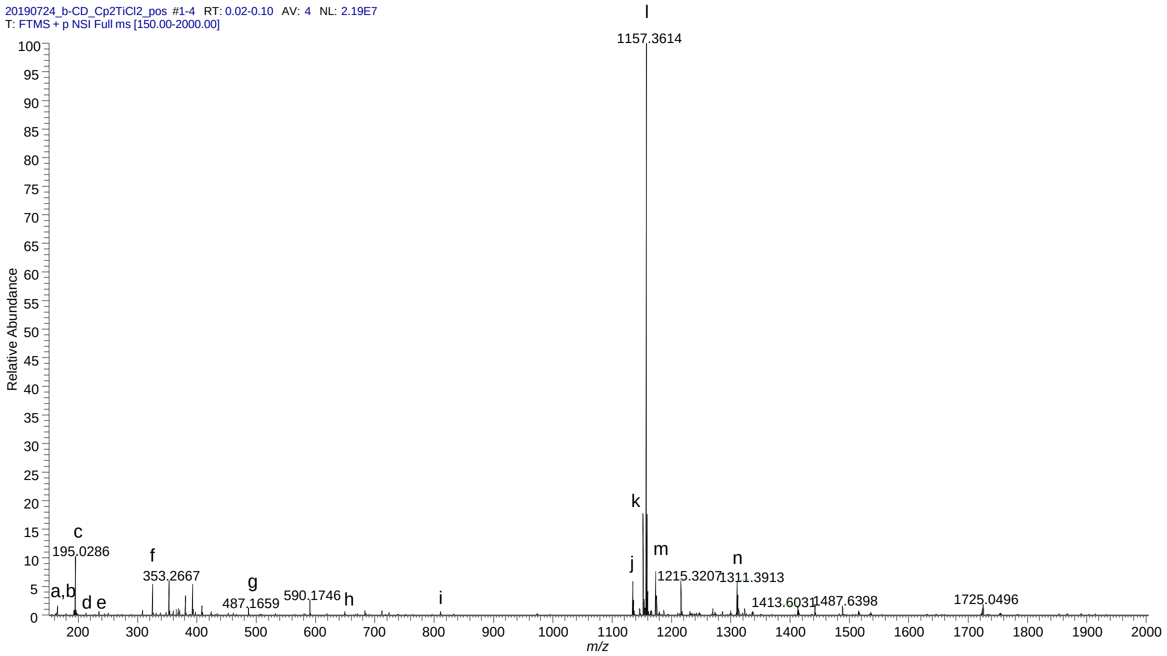

	m/z experimental	Name	*m/z* calculation	Error
a	163.0603	$[glc+H]^+$	163.0601	1.2 ppm
b	165.0027	$[CpTi(OH)_2+(H_2O)]^+$	165.0026	0.6 ppm
c	195.0286	$[Cp_2Ti(OH)]^+$	195.0284	1.0 ppm
d	213.0392	$[Cp_2Ti(OH)+(H_2O)]^+$	213.0390	0.9 ppm
e	235.0213	$[Cp_2Ti(OH)_2+Na]^+$	235.0209	1.7 ppm
f	325.1134	$[glc_2+H]^+$	325.1129	1.5 ppm
g	487.1659	$[glc_3+H]^+$	487.1657	0.4 ppm
h	649.2191	$[glc_4+H]^+$	649.2186	0.8 ppm
i	811.2728	$[glc_5+H]^+$	811.2714	1.7 ppm
j	1135.3807	$[\beta CD+H]^+$	1135.3770	3.3 ppm
k	1152.4073	$[\beta CD+NH_4]^+$	1152.4036	3.2 ppm
l	1157.3614	$[\beta CD+Na]^+$	1157.3590	2.1 ppm
m	1173.3357	$[\beta CD+K]^+$	1173.3329	2.4 ppm
n	1311.3913	$[\beta CD+Cp_2Ti-H]^+$	1311.3876	2.8 ppm

Table S11. Tandem mass spectrum of the β-cyclodextrin/titanocene dichloride mixture in 50/50 H_2O/MeCN. Precursor ion: $[\beta CD+Cp_2Ti-H]^+$ *m/z* 1311.40, HCD at 20% NCE (47 eV), 100% = 8.82E5.

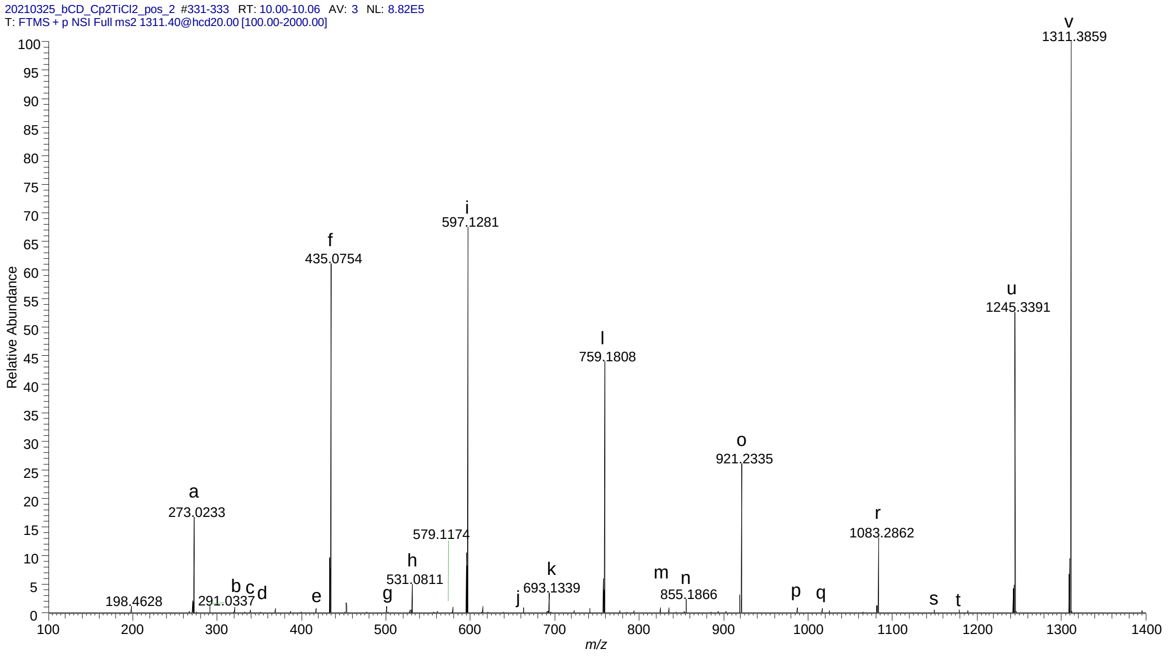

	m/z experimental	Name	*m/z* calculation	Error
a	273.0233	$[glc_1+CpTi-2H]^+$	273.0237	-1.5 ppm
b	321.0594	$[glc-H_2O+Cp_2Ti-H]^+$	321.0601	-2.2 ppm
c	339.0699	$[glc_1+Cp_2Ti-H]^+$	339.0706	-2.1 ppm
d	369.0287	$[glc_2+Ti-3H]^+$	369.0296	-2.4 ppm
e	417.0649	$[glc_2-H_2O+CpTi-2H]^+$	417.0660	-2.6 ppm
f	435.0754	$[glc_2+CpTi-2H]^+$	435.0765	-2.5 ppm
g	501.1221	$[glc_2+Cp_2Ti-H]^+$	501.1235	-2.8 ppm
h	531.0811	$[glc_3+Ti-3H]^+$	531.0824	-2.4 ppm
i	597.1281	$[glc_3+CpTi-2H]^+$	597.1293	-2.0 ppm
j	663.1747	$[glc_3+Cp_2Ti-H]^+$	663.1763	-2.4 ppm
k	693.1339	$[glc_4+Ti-3H]^+$	693.1352	-1.9 ppm
l	759.1808	$[glc_4+CpTi-2H]^+$	759.1822	-1.8 ppm
m	825.2277	$[glc_4+Cp_2Ti-H]^+$	825.2291	-1.7 ppm
n	855.1866	$[glc_5+Ti-3H]^+$	855.1880	-1.6 ppm
o	921.2335	$[glc_5+CpTi-2H]^+$	921.2350	-1.6 ppm
p	987.2802	$[glc_5+Cp_2Ti-H]^+$	987.2819	-1.7 ppm
q	1017.2391	$[glc_6+Ti-3H]^+$	1017.2409	-1.8 ppm
r	1083.2862	$[glc_6+CpTi-2H]^+$	1083.2878	-1.5 ppm
s	1149.3330	$[glc_6+Cp_2Ti-H]^+$	1149.3348	-1.6 ppm
t	1179.2922	$[\beta CD+Ti-3H]^+$	1179.2937	-1.3 ppm
u	1245.3391	$[\beta CD+CpTi-2H]^+$	1245.3406	-1.2 ppm
v	1311.3859	$[\beta CD+Cp_2Ti-H]^+$	1311.3876	-1.3 ppm

Table S12. Full scan mass spectrum of the sucrose/titanocene dichloride mixture in 50/50 H_2O/MeCN (100% = 3.67E7).

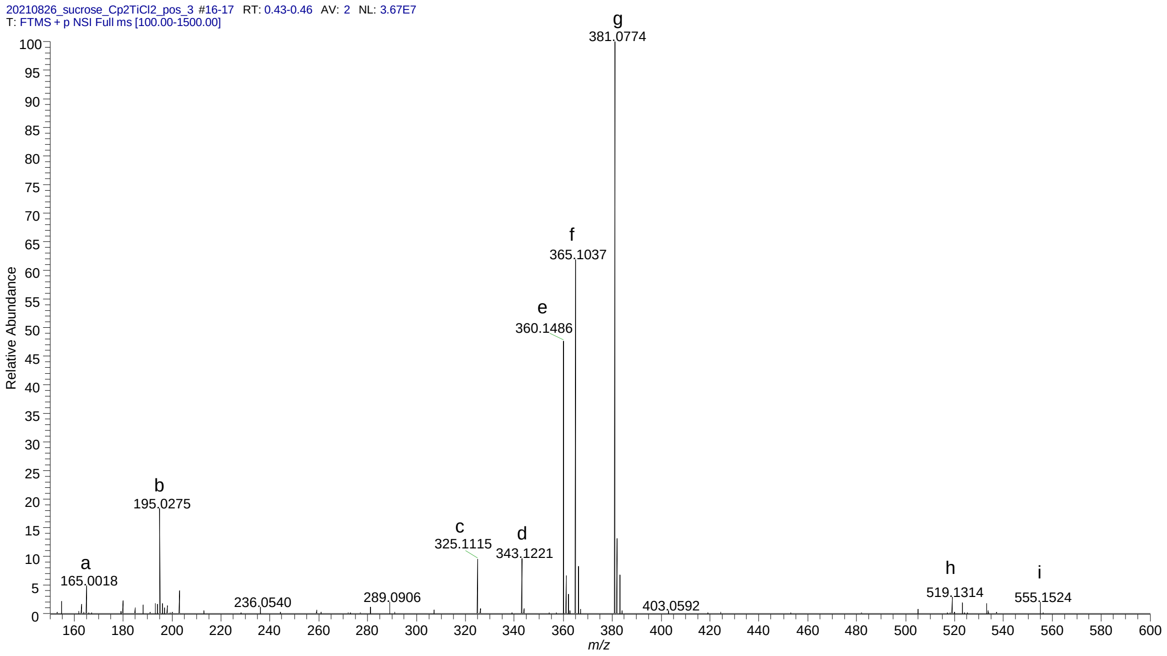

	m/z experimental	Name	*m/z* calculation	Error
a	165.0018	$[CpTi(OH)_2(H_2O)]^+$	165.0026	-4.8 ppm
b	195.0275	$[Cp_2Ti(OH)]^+$	195.0284	-4.5 ppm
c	325.1115	$[suc\text{-}H_2O\text{+}H]^+$	325.1129	-4.2 ppm
d	343.1221	$[suc\text{+}H]^+$	343.1235	-4.2 ppm
e	360.1486	$[suc\text{+}NH_4]^+$	360.1500	-4.0 ppm
f	365.1037	$[suc\text{+}Na]^+$	365.1054	-4.8 ppm
g	381.0774	$[suc\text{+}K]^+$	381.0794	-5.2 ppm
h	519.1314	$[suc\text{+}Cp_2Ti\text{-}H]^+$	519.1340	-5.1 ppm
i	555.1524	$[suc\text{+}Cp_2Ti(OH)(H_2O)]^+$	555.1552	-5.0 ppm

Table S13. Tandem mass spectrum of the sucrose/titanocene dichloride mixture in 50/50 H_2O/MeCN. Precursor ion: $[suc+Cp_2Ti-H]^+$ *m/z* 519.20, HCD at 19% NCE (17 eV), 100% = 2.17E5.

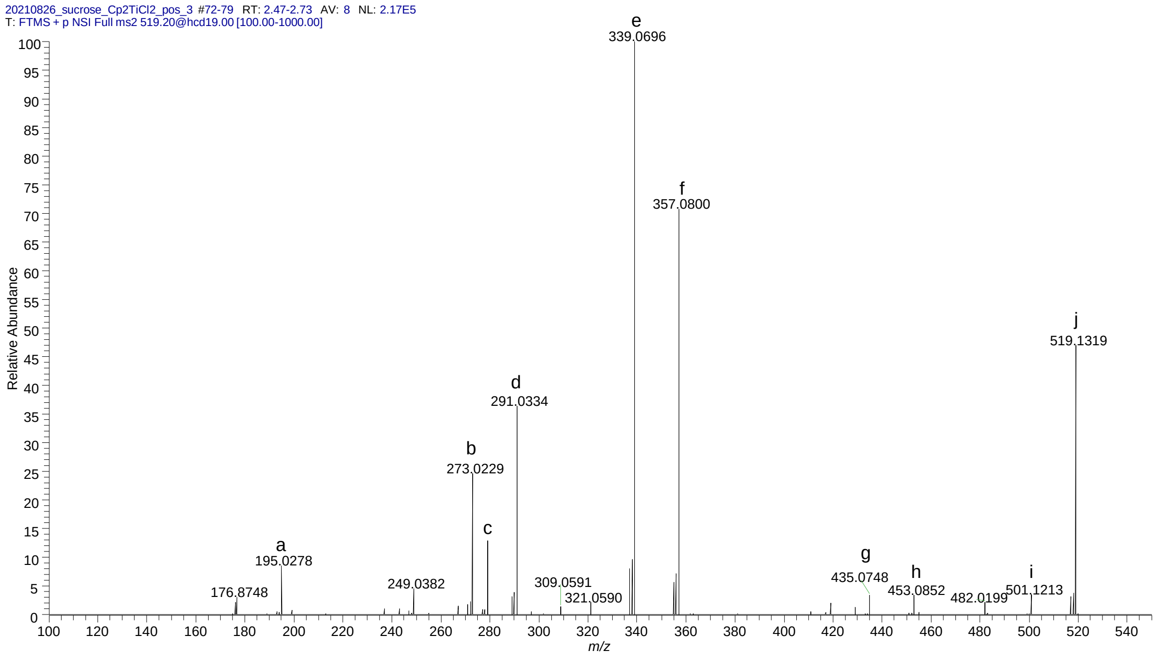

	m/z experimental	Name	*m/z* calculation	Error
a	195.0278	$[Cp_2Ti(OH)]^+$	195.0284	-3.1 ppm
b	273.0229	$[C_{11}H_{13}O_5Ti]^+$	273.0237	-2.9 ppm
c	279.0487	$[C_{14}H_{15}O_3Ti]^+$	279.0495	-3.0 ppm
d	291.0334	$[C_{11}H_{15}O_6Ti]^+$	291.0343	-3.0 ppm
e	339.0696	$[C_6H_{10}O_5+Cp_2Ti-H]^+$	339.0706	-3.2 ppm
f	357.0800	$[C_6H_{12}O_6+Cp_2Ti-H]^+$	357.0812	-3.4 ppm
g	435.0748	$[suc-H_2O+CpTi-2H]^+$	435.0765	-3.9 ppm
h	453.0852	$[suc+CpTi-2H]^+$	453.0871	-4.2 ppm
i	501.1213	$[suc-H_2O+Cp_2Ti-H]^+$	501.1235	-4.4 ppm
j	519.1319	$[suc+Cp_2Ti-H]^+$	519.1340	-4.1 ppm

Table S14. Full scan mass spectrum of the maltose/titanocene dichloride mixture in 50/50 H_2O/MeCN (100% = 1.06E7).

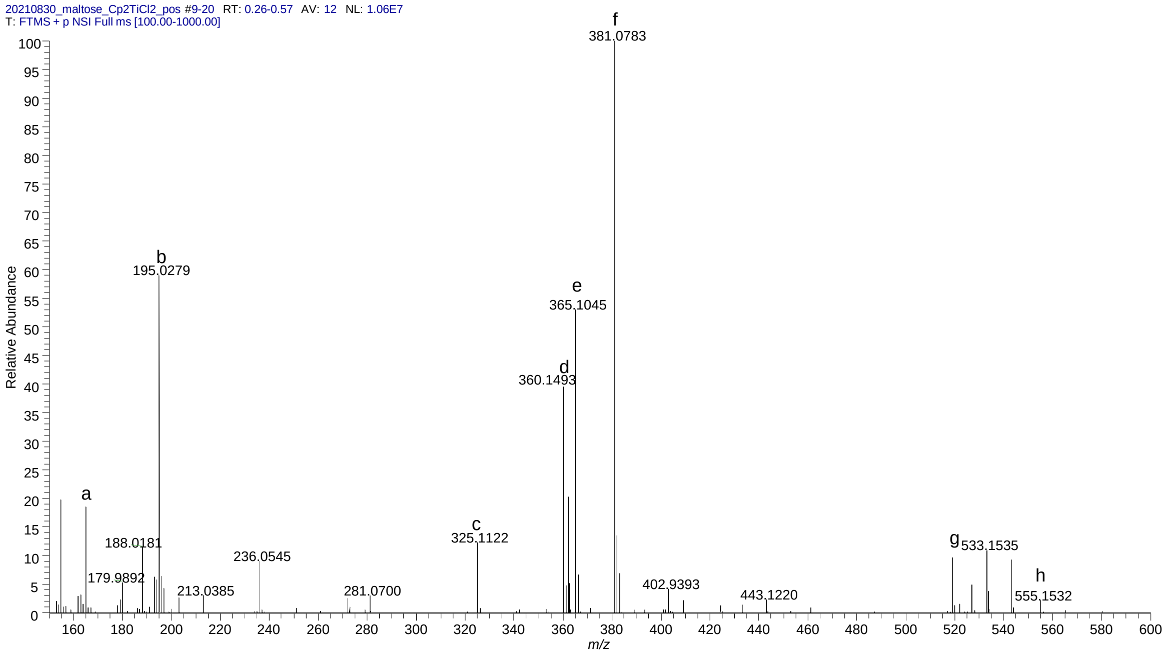

	m/z experimental	Name	*m/z* calculation	Error
a	165.0021	$[CpTi(OH)_2(H_2O)]^+$	165.0026	-2.7 ppm
b	195.0279	$[Cp_2Ti(OH)]^+$	195.0284	-2.5 ppm
c	325.1122	$[maltose-H_2O+H]^+$	325.1129	-2.3 ppm
d	360.1493	$[maltose+NH_4]^+$	360.1500	-2.0 ppm
e	365.1045	$[maltose+Na]^+$	365.1054	-2.6 ppm
f	381.0783	$[maltose+K]^+$	381.0794	-2.8 ppm
g	519.1325	$[maltose+Cp_2Ti-H]^+$	519.1340	-3.1 ppm
h	555.1532	$[maltose+Cp_2Ti(OH)(H_2O)]^+$	555.1552	-3.6 ppm

Table S15. Tandem mass spectrum of the maltose/titanocene dichloride mixture in 50/50 H_2O/MeCN. Precursor ion: $[maltose+Cp_2Ti-H]^+$ *m/z* 519.20, HCD at 20% NCE (18 eV), 100% = 2.20E5.

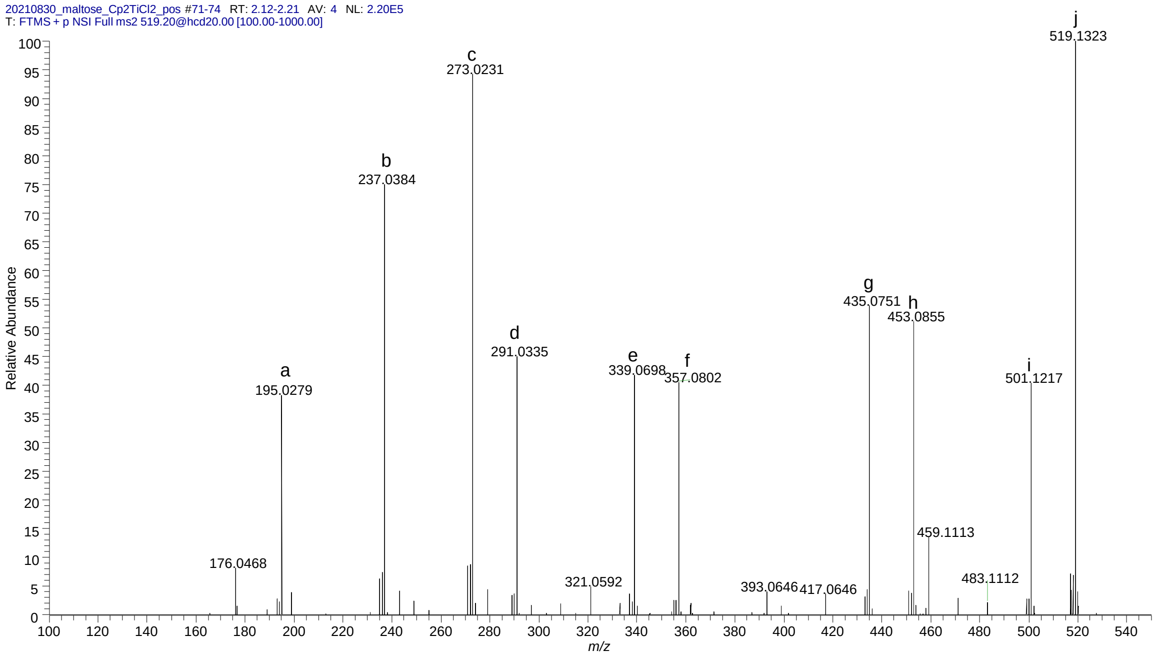

	m/z experimental	Name	*m/z* calculation	Error
a	195.0279	$[Cp_2Ti(OH)]^+$	195.0284	-2.6 ppm
b	237.0384	$[C_{12}H_{13}O_2Ti]^+$	237.0390	-2.4 ppm
c	273.0231	$[C_{11}H_{13}O_5Ti]^+$	273.0237	-2.3 ppm
d	291.0335	$[C_{11}H_{15}O_6Ti]^+$	291.0343	-2.5 ppm
e	339.0698	$[C_6H_{10}O_5+Cp_2Ti-H]^+$	339.0706	-2.6 ppm
f	357.0802	$[C_6H_{12}O_6+Cp_2Ti-H]^+$	357.0812	-2.8 ppm
g	435.0751	$[maltose-H_2O+CpTi-2H]^+$	435.0765	-3.2 ppm
h	453.0855	$[maltose+CpTi-2H]^+$	453.0871	-3.4 ppm
i	501.1217	$[maltose-H_2O+Cp_2Ti-H]^+$	501.1235	-3.6 ppm
j	519.1323	$[maltose+Cp_2Ti-H]^+$	519.1340	-3.4 ppm

Table S16. Full scan mass spectrum of DMβ-cyclodextrin in 50/50 H_2O/MeCN +1% FA (100% = 2.94E7).

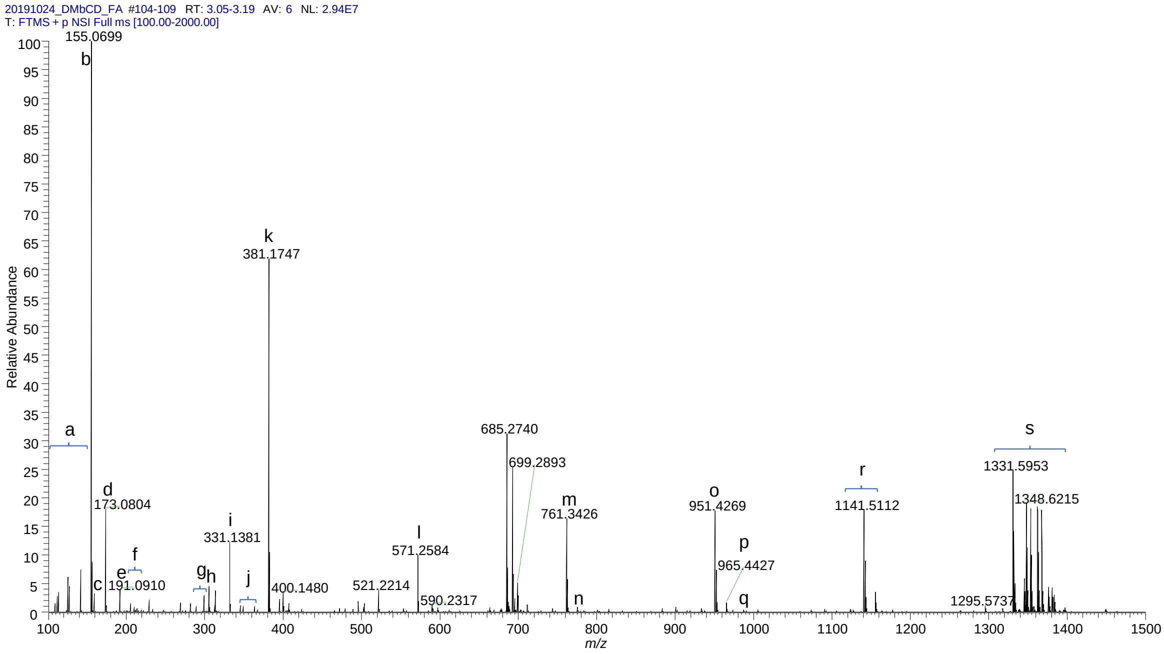

	m/z experimental	Name	*m/z* calculation	Error
a	109.0280	[DMglc-H_2O-2CH_3OH+H]$^+$	109.0284	-3.7 ppm
	125.0593	[DMglc-2H_2O-CH_2O+H]$^+$	125.0597	-3.2 ppm
	127.0386	[DMglc-2CH_3OH+H]$^+$	127.0390	-3.1 ppm
	141.0543	[DMglc-H_2O-CH_3OH+H]$^+$	141.0546	-2.1 ppm
b	155.0699	[DMglc-2H_2O+H]$^+$	155.0703	-2.6 ppm
c	159.0648	[DMglc-CH_3OH+H]$^+$	159.0652	-2.5 ppm
d	173.0804	[DMglc-H_2O+H]$^+$	173.0808	-2.3 ppm
e	191.0910	[DMglc+H]$^+$	191.0914	-2.1 ppm
f	205.1067	[DMglc+CH_2+H]$^+$	205.1071	-2.0 ppm
	213.0729	[DMglc+Na]$^+$	213.0733	-1.9 ppm
g	281.1015	[DMglc_2-2H_2O-2CH_3OH+H]$^+$	281.1020	-1.8 ppm
	299.1120	[DMglc_2-H_2O-2CH_3OH+H]$^+$	299.1125	-1.7 ppm
h	313.1276	[DMglc_2-2H_2O-CH_3OH+H]$^+$	313.1282	-1.9 ppm
i	331.1381	[DMglc_2-H_2O-CH_3OH+H]$^+$	331.1387	-1.8 ppm
j	349.1486	[DMglc_2-CH_3OH+H]$^+$	349.1493	-2.0 ppm
	363.1642	[DMglc_2-H_2O+H]$^+$	363.1650	-2.2 ppm
k	381.1747	[DMglc_2+H]$^+$	381.1755	-2.1 ppm
l	571.2584	[DMglc_3+H]$^+$	571.2596	-2.1 ppm
m	761.3426	[DMglc_4+H]$^+$	761.3438	-1.6 ppm
n	775.3581	[DMglc_4+CH_2+H]$^+$	775.3594	-1.7 ppm
o	951.4269	[DMglc_5+H]$^+$	951.4279	-1.1 ppm
p	965.4427	[DMglc_5+CH_2+H]$^+$	965.4435	-0.8 ppm
q	987.4241	[DMglc_5+CH_2+Na]$^+$	987.4255	-1.4 ppm
r	1127.4951	[DMglc_6-CH_2+H]$^+$	1127.4964	-1.2 ppm
	1141.5112	[DMglc_6+H]$^+$	1141.5120	-0.7 ppm
	1144.5199	[DMglc_6-CH_2+NH_4]$^+$	1144.5229	-2.6 ppm
	1155.5265	[DMglc_6+CH_2+H]$^+$	1155.5277	-1.0 ppm
s	1317.5796	[DMβCD-CH_2+H]$^+$	1317.5805	-0.7 ppm
	1331.5953	[DMβCD+H]$^+$	1331.5961	-0.6 ppm
	1334.6044	[DMβCD-CH_2+NH_4]$^+$	1334.6070	-1.9 ppm
	1345.6108	[DMβCD+CH_2+H]$^+$	1345.6112	-0.3 ppm

1348.6215	$[DM\beta CD+NH_4]^+$	1348.6227	-0.9 ppm
1353.5766	$[DM\beta CD+Na]^+$	1353.5781	-1.1 ppm
1359.6260	$[DM\beta CD+2CH_2+H]^+$	1359.6274	-1.0 ppm
1362.6368	$[DM\beta CD+CH_2+NH_4]^+$	1362.6383	-1.1 ppm
1367.5921	$[DM\beta CD+CH_2+Na]^+$	1367.5937	-1.2 ppm
1369.5504	$[DM\beta CD+K]^+$	1369.5520	-1.2 ppm
1376.6523	$[DM\beta CD+2CH_2+NH_4]^+$	1376.6540	-1.2 ppm
1381.6075	$[DM\beta CD+2CH_2+Na]^+$	1381.6094	-1.4 ppm
1383.5661	$[DM\beta CD+CH_2+K]^+$	1383.5677	-1.2 ppm
1395.6231	$[DM\beta CD+3CH_2+Na]^+$	1395.6250	-1.4 ppm
1397.5815	$[DM\beta CD+2CH_2+K]^+$	1397.5833	-1.3 ppm

Table S17. Full scan mass spectrum of TMβ-cyclodextrin in 50/50 H_2O/MeCN (100% = 4.86E6).

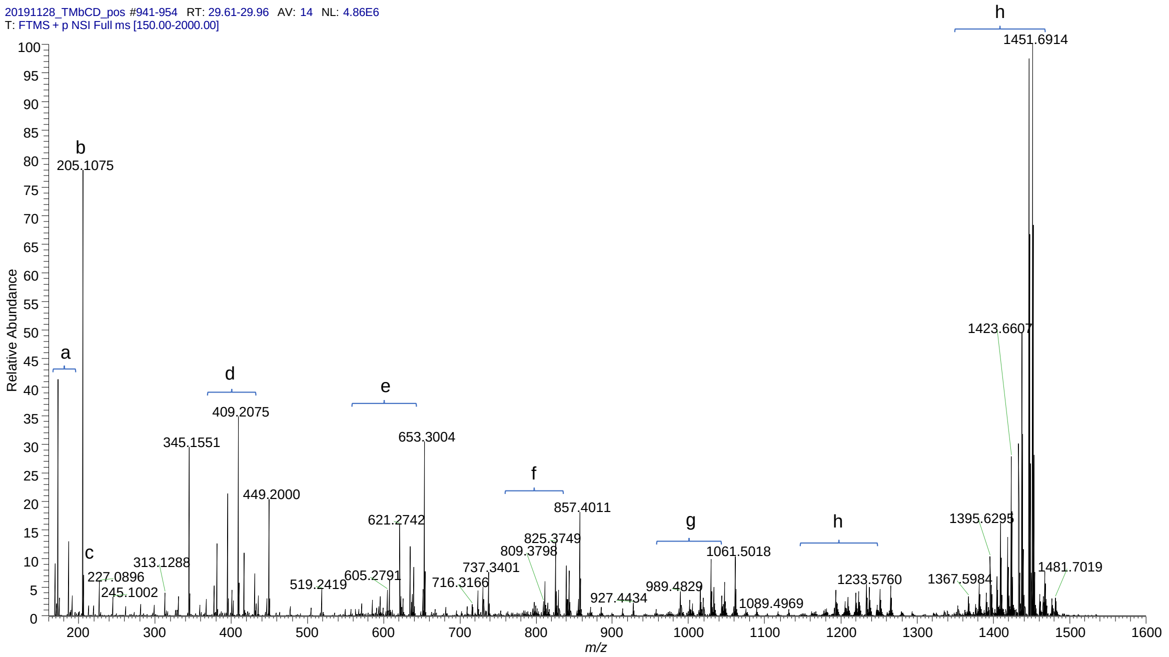

	m/z experimental	Name	*m/z* calculation	Error
a	173.0811	$[TMglc-CH_3OH+H]^+$	173.0808	1.7 ppm
	187.0968	$[TMglc-H_2O+H]^+$	187.0965	1.6 ppm
	191.0918	$[TMglc-CH_2+H]^+$	191.0914	2.1 ppm
b	205.1075	$[TMglc+H]^+$	205.1071	2.0 ppm
c	213.0738	$[TMglc-CH_2+Na]^+$	213.0733	2.3 ppm
d	353.1448	$[TMglc_2-4CH_2+H]^+$	353.1442	1.7 ppm
	367.1606	$[TMglc_2-3CH_2+H]^+$	367.1599	1.9 ppm
	381.1763	$[TMglc_2-2CH_2+H]^+$	381.1755	2.1 ppm
	389.1424	$[TMglc_2-3CH_2+Na]^+$	389.1418	1.5 ppm
	395.1919	$[TMglc_2-CH_2+H]^+$	395.1912	1.8 ppm
	403.1582	$[TMglc_2-2CH_2+Na]^+$	403.1575	1.7 ppm
	409.2075	$[TMglc_2+H]^+$	409.2068	1.7 ppm
	417.1738	$[TMglc_2-CH_2+Na]^+$	417.1731	1.7 ppm
	431.1894	$[TMglc_2+Na]^+$	431.1888	1.4 ppm
e	557.2448	$[TMglc_3-4CH_2+H]^+$	557.2440	1.4 ppm
	571.2605	$[TMglc_3-3CH_2+H]^+$	571.2596	1.6 ppm
	579.2269	$[TMglc_3-4CH_2+Na]^+$	579.2259	1.7 ppm
	585.2763	$[TMglc_3-2CH_2+H]^+$	585.2753	1.7 ppm
	593.2425	$[TMglc_3-3CH_2+Na]^+$	593.2416	1.5 ppm
	599.2919	$[TMglc_3-CH_2+H]^+$	599.2909	1.7 ppm
	607.2584	$[TMglc_3-2CH_2+Na]^+$	607.2572	2.0 ppm
	621.2742	$[TMglc_3-CH_2+Na]^+$	621.2729	2.1 ppm
	635.2899	$[TMglc_3+Na]^+$	635.2885	2.2 ppm
f	761.3456	$[TMglc_4-4CH_2+H]^+$	761.3438	2.4 ppm
	769.3120	$[TMglc_4-5CH_2+Na]^+$	769.3101	2.5 ppm
	775.3613	$[TMglc_4-3CH_2+H]^+$	775.3594	2.5 ppm
	783.3276	$[TMglc_4-4CH_2+Na]^+$	783.3257	2.4 ppm
	789.3771	$[TMglc_4-2CH_2+H]^+$	789.3751	2.5 ppm
	797.3434	$[TMglc_4-3CH_2+Na]^+$	797.3414	2.5 ppm
	803.3928	$[TMglc_4-CH_2+H]^+$	803.3907	2.6 ppm
	811.3591	$[TMglc_4-2CH_2+Na]^+$	811.3570	2.6 ppm

	817.4084	$[TMglc_4+H]^+$	817.4064	2.4 ppm
	825.3749	$[TMglc_4-CH_2+Na]^+$	825.3727	2.7 ppm
	839.3905	$[TMglc_4+Na]^+$	839.3883	2.6 ppm
g	959.3972	$[TMglc_5-6CH_2+Na]^+$	959.3942	3.1 ppm
	973.4124	$[TMglc_5-5CH_2+Na]^+$	973.4098	2.7 ppm
	987.4283	$[TMglc_5-4CH_2+Na]^+$	987.4255	2.8 ppm
	993.4776	$[TMglc_5-2CH_2+H]^+$	993.4748	2.8 ppm
	1001.4442	$[TMglc_5-3CH_2+Na]^+$	1001.4411	3.1 ppm
	1007.4935	$[TMglc_5-CH_2+H]^+$	1007.4905	3.0 ppm
	1015.4598	$[TMglc_5-2CH_2+Na]^+$	1015.4568	3.0 ppm
	1021.5092	$[TMglc_5+H]^+$	1021.5061	3.0 ppm
	1029.4756	$[TMglc_5-CH_2+Na]^+$	1029.4724	3.1 ppm
	1043.4911	$[TMglc_5+Na]^+$	1043.4881	2.9 ppm
h	1149.4823	$[TMglc_6-7CH_2+Na]^+$	1149.4783	3.5 ppm
	1163.4976	$[TMglc_6-6CH_2+Na]^+$	1163.4940	3.1 ppm
	1177.5133	$[TMglc_6-5CH_2+Na]^+$	1177.5096	3.1 ppm
	1183.5631	$[TMglc_6-3CH_2+H]^+$	1183.5590	3.5 ppm
	1191.5291	$[TMglc_6-4CH_2+Na]^+$	1191.5253	3.2 ppm
	1197.5789	$[TMglc_6-2CH_2+H]^+$	1197.5746	3.6 ppm
	1205.5447	$[TMglc_6-3CH_2+Na]^+$	1205.5409	3.2 ppm
	1211.5943	$[TMglc_6-CH_2+H]^+$	1211.5903	3.3 ppm
	1219.5603	$[TMglc_6-2CH_2+Na]^+$	1219.5566	3.0 ppm
	1225.6098	$[TMglc_6+H]^+$	1225.6059	3.2 ppm
	1233.5760	$[TMglc_6-CH_2+Na]^+$	1233.5722	3.1 ppm
	1247.5913	$[TMglc_6+Na]^+$	1247.5879	2.7 ppm
i	1348.6275	$[TM\beta CD-7CH_2+NH_4]^+$	1348.6227	3.6 ppm
	1353.5828	$[TM\beta CD-7CH_2+Na]^+$	1353.5781	3.5 ppm
	1359.6325	$[TM\beta CD-5CH_2+H]^+$	1359.6274	3.8 ppm
	1362.6433	$[TM\beta CD-6CH_2+NH_4]^+$	1362.6383	3.7 ppm
	1367.5984	$[TM\beta CD-6CH_2+Na]^+$	1367.5937	3.4 ppm
	1373.6481	$[TM\beta CD-4CH_2+H]^+$	1373.6431	3.6 ppm
	1376.6588	$[TM\beta CD-5CH_2+NH_4]^+$	1376.6540	3.5 ppm
	1381.6141	$[TM\beta CD-5CH_2+Na]^+$	1381.6094	3.4 ppm
	1387.6632	$[TM\beta CD-3CH_2+H]^+$	1387.6587	3.2 ppm
	1390.6744	$[TM\beta CD-4CH_2+NH_4]^+$	1390.6696	3.5 ppm
	1395.6295	$[TM\beta CD-4CH_2+Na]^+$	1395.6250	3.2 ppm
	1401.6792	$[TM\beta CD-2CH_2+H]^+$	1401.6744	3.4 ppm
	1404.6899	$[TM\beta CD-3CH_2+NH_4]^+$	1404.6853	3.3 ppm
	1409.6453	$[TM\beta CD-3CH_2+Na]^+$	1409.6407	3.3 ppm
	1415.6947	$[TM\beta CD-CH_2+H]^+$	1415.6900	3.3 ppm
	1418.7055	$[TM\beta CD-2CH_2+NH_4]^+$	1418.7009	3.2 ppm
	1423.6607	$[TM\beta CD-2CH_2+Na]^+$	1423.6563	3.1 ppm
	1429.7100	$[TM\beta CD+H]^+$	1429.7057	3.0 ppm
	1432.7210	$[TM\beta CD-CH_2+NH_4]^+$	1432.7166	3.1 ppm
	1437.6762	$[TM\beta CD-CH_2+Na]^+$	1437.6720	2.9 ppm
	1439.6319	$[TM\beta CD-2CH_2+K]^+$	1439.6303	1.1 ppm
	1446.7364	$[TM\beta CD+NH_4]^+$	1446.7322	2.9 ppm
	1451.6914	$[TM\beta CD+Na]^+$	1451.6876	2.6 ppm
	1453.6497	$[TM\beta CD-CH_2+K]^+$	1453.6459	2.6 ppm
	1467.6652	$[TM\beta CD+K]^+$	1467.6616	2.5 ppm

Table S18. Full scan mass spectrum of the DMβ-cyclodextrin/phenylalanine mixture in 50/50 H_2O/MeCN (100% = 1.08E7).

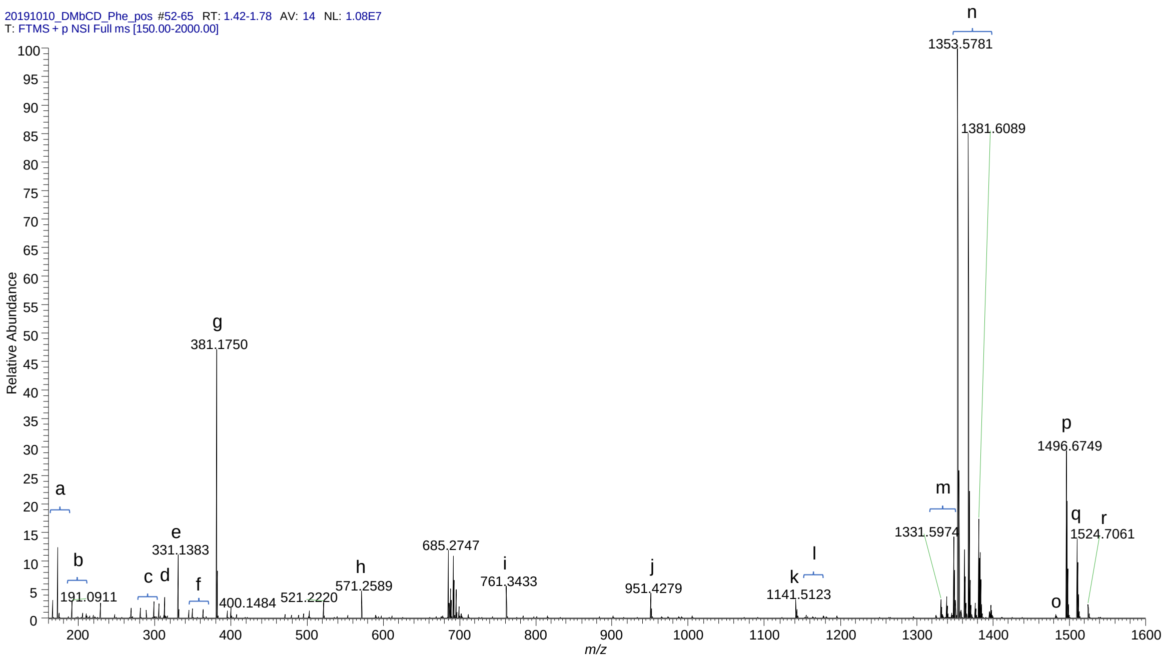

	m/z experimental	Name	*m/z* calculation	Error
a	166.0859	$[Phe+H]^+$	166.0863	-2.4 ppm
	173.0805	$[DMglc-H_2O+H]^+$	173.0808	-1.7 ppm
b	191.0911	$[DMglc+H]^+$	191.0914	-1.6 ppm
	205.1068	$[DMglc+CH_2+H]^+$	205.1071	-1.5 ppm
c	281.1016	$[DMglc_2-2H_2O-2CH_3OH+H]^+$	281.1020	-1.4 ppm
	299.1122	$[DMglc_2-H_2O-2CH_3OH+H]^+$	299.1125	-1.0 ppm
d	313.1278	$[DMglc_2-2H_2O-CH_3OH+H]^+$	313.1282	-1.3 ppm
e	331.1383	$[DMglc_2-H_2O-CH_3OH+H]^+$	331.1387	-1.2 ppm
f	349.1488	$[DMglc_2-CH_3OH+H]^+$	349.1493	-1.4 ppm
	363.1645	$[DMglc_2-H_2O+H]^+$	363.1650	-1.4 ppm
g	381.1750	$[DMglc_2+H]^+$	381.1755	-1.3 ppm
h	571.2589	$[DMglc_3+H]^+$	571.2596	-1.2 ppm
i	761.3433	$[DMglc_4+H]^+$	761.3438	-0.7 ppm
j	951.4279	$[DMglc_5+H]^+$	951.4279	0.0 ppm
k	1141.5123	$[DMglc_6+H]^+$	1141.5120	0.3 ppm
l	1155.5278	$[DMglc_6+CH_2+H]^+$	1155.5277	0.1 ppm
	1177.5099	$[DMglc_6+CH_2+Na]^+$	1177.5096	0.3 ppm
m	1325.5481	$[DM\beta CD-2CH_2+Na]^+$	1325.5468	1.0 ppm
	1331.5974	$[DM\beta CD+H]^+$	1331.5961	1.0 ppm
	1334.6074	$[DM\beta CD-CH_2+NH_4]^+$	1334.6070	0.3 ppm
	1339.5635	$[DM\beta CD-CH_2+Na]^+$	1339.5624	0.8 ppm
	1345.6128	$[DM\beta CD+CH_2+H]^+$	1345.6112	1.2 ppm
	1348.6238	$[DM\beta CD+NH_4]^+$	1348.6227	0.8 ppm
n	1353.5781	$[DM\beta CD+Na]^+$	1353.5781	0.0 ppm
	1362.6388	$[DM\beta CD+CH_2+NH_4]^+$	1362.6383	0.4 ppm
	1367.5931	$[DM\beta CD+CH_2+Na]^+$	1367.5937	-0.4 ppm
	1369.5525	$[DM\beta CD+K]^+$	1369.5520	0.4 ppm
	1376.6535	$[DM\beta CD+2CH_2+NH_4]^+$	1376.6540	-0.4 ppm
	1381.6089	$[DM\beta CD+2CH_2+Na]^+$	1381.6094	-0.4 ppm
	1383.5672	$[DM\beta CD+CH_2+K]^+$	1383.5677	-0.4 ppm

	1395.6243	[DMβCD+3CH_2+Na]$^+$	1395.6250	-0.5 ppm
	1397.5826	[DMβCD+2CH_2+K]$^+$	1397.5833	-0.5 ppm
o	1482.6595	[DMβCD-CH_2+Phe+H]$^+$	1482.6595	0.0 ppm
p	1496.6749	[DMβCD+Phe+H]$^+$	1496.6751	-0.1 ppm
	1499.6838	[DMβCD-CH_2+Phe+NH_4]$^+$	1499.6860	-1.5 ppm
q	1510.6906	[DMβCD+CH_2+Phe+H]$^+$	1510.6908	-0.1 ppm
	1513.6995	[DMβCD+Phe+NH_4]$^+$	1513.7017	-1.5 ppm
r	1524.7061	[DMβCD+2CH_2+Phe+H]$^+$	1524.7064	-0.2 ppm

Table S19. Tandem mass spectrum of the DMβ-cyclodextrin/phenylalanine mixture in 50/50 H_2O/MeCN. Precursor ion: [DMβCD+Phe+H]$^+$ *m/z* 1496.70, HCD at 15% NCE (40 eV), 100% = 1.08E6.

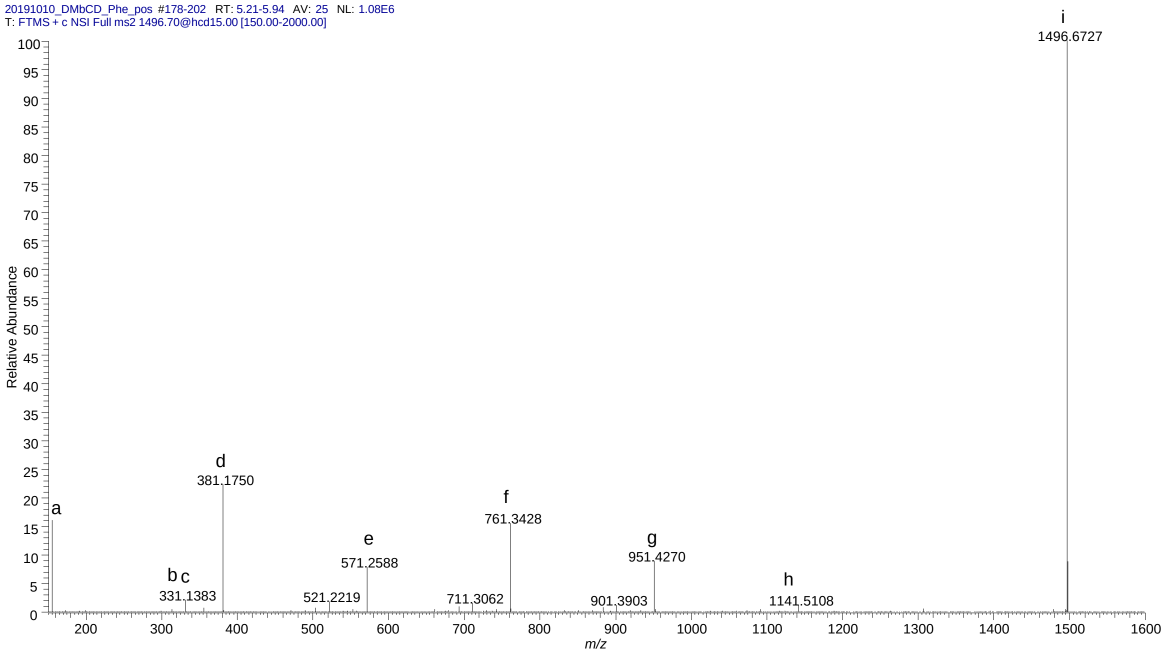

	m/z experimental	Name	*m/z* calculation	Error
a	155.0700	[DMglc-2H_2O+H]$^+$	155.0703	-1.9 ppm
b	313.1278	[$DMglc_2$-2H_2O-CH_3OH+H]$^+$	313.1282	-1.3 ppm
c	331.1383	[$DMglc_2$-H_2O-CH_3OH+H]$^+$	331.1387	-1.2 ppm
d	381.1750	[$DMglc_2$+H]$^+$	381.1755	-1.3 ppm
e	571.2588	[$DMglc_3$+H]$^+$	571.2596	-1.4 ppm
f	761.3428	[$DMglc_4$+H]$^+$	761.3438	-1.3 ppm
g	951.4270	[$DMglc_5$+H]$^+$	951.4279	-0.9 ppm
h	1141.5108	[$DMglc_6$+H]$^+$	1141.5120	-1.1 ppm
i	1496.6727	[DMβCD+Phe+H]$^+$	1496.6751	-1.6 ppm

Table S20. Full scan mass spectrum of the DMβ-cyclodextrin/titanocene dichloride mixture in 50/50 H_2O/MeCN (100% = 7.57E7).

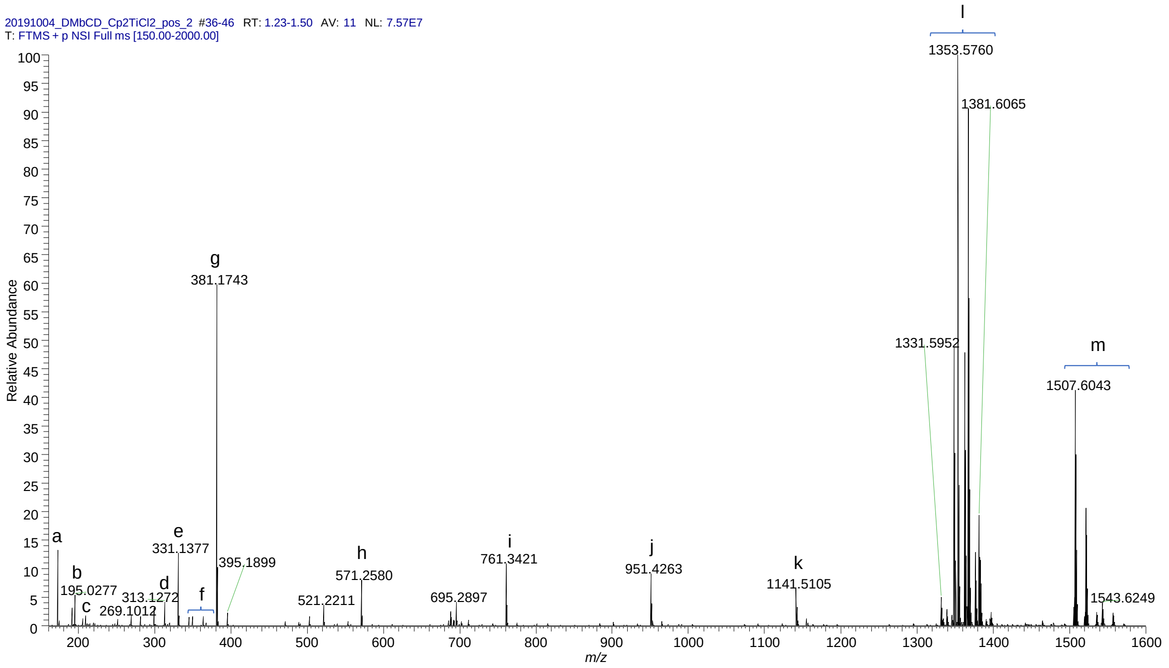

	m/z experimental	Name	*m/z* calculation	Error
a	173.0802	[DMglc-H_2O+H]$^+$	173.0808	-3.5 ppm
	191.0908	[DMglc+H]$^+$	191.0914	-3.1 ppm
b	195.0277	[Cp_2Ti(OH)]$^+$	195.0284	-3.6 ppm
	205.1064	[DMglc+CH_2+H]$^+$	205.1071	-3.4 ppm
c	281.1011	[$DMglc_2$-2H_2O-2CH_3OH+H]$^+$	281.1020	-3.2 ppm
	299.1116	[$DMglc_2$-H_2O-2CH_3OH+H]$^+$	299.1125	-3.0 ppm
d	313.1272	[$DMglc_2$-2H_2O-CH_3OH+H]$^+$	313.1282	-3.2 ppm
e	331.1377	[$DMglc_2$-H_2O-CH_3OH+H]$^+$	331.1387	-3.0 ppm
	349.1482	[$DMglc_2$-CH_3OH+H]$^+$	349.1493	-3.2 ppm
f	363.1639	[$DMglc_2$-H_2O+H]$^+$	363.1650	-3.0 ppm
	367.1008	[DMglc+Cp_2Ti-H]$^+$	367.1019	-3.0 ppm
	367.1587	[$DMglc_2$-CH_2+H]$^+$	367.1599	-3.3 ppm
g	381.1743	[$DMglc_2$+H]$^+$	381.1755	-3.1 ppm
h	571.2580	[$DMglc_3$+H]$^+$	571.2596	-2.8 ppm
i	761.3421	[$DMglc_4$+H]$^+$	761.3438	-2.2 ppm
	775.3576	[$DMglc_4$+CH_2+H]$^+$	775.3594	-2.3 ppm
j	951.4263	[$DMglc_5$+H]$^+$	951.4279	-1.7 ppm
	965.4419	[$DMglc_5$+CH_2+H]$^+$	965.4435	-1.7 ppm
k	1141.5105	[$DMglc_6$+H]$^+$	1141.5120	-1.3 ppm
	1155.5259	[$DMglc_6$+CH_2+H]$^+$	1155.5277	-1.6 ppm
	1325.5461	[DMβCD-2CH_2+Na]$^+$	1325.5468	-0.5 ppm
	1331.5952	[DMβCD+H]$^+$	1331.5961	-0.7 ppm
	1334.6058	[DMβCD-CH_2+NH_4]$^+$	1334.6070	-0.9 ppm
	1339.5616	[DMβCD-CH_2+Na]$^+$	1339.5624	-0.6 ppm
l	1345.6107	[DMβCD+CH_2+H]$^+$	1345.6112	-0.4 ppm
	1348.6215	[DMβCD+NH_4]$^+$	1348.6227	-0.9 ppm
	1353.5760	[DMβCD+Na]$^+$	1353.5781	-1.6 ppm
	1358.6069	[DMβCD+CH_2+Ti-4H+NH_4]$^+$	1358.6070	-0.1 ppm
	1362.6363	[DMβCD+CH_2+NH_4]$^+$	1362.6383	-1.5 ppm
	1367.5910	[DMβCD+CH_2+Na]$^+$	1367.5937	-2.0 ppm

	1369.5501	[DMβCD+K]$^+$	1369.5520	-1.4 ppm
	1376.6515	[DMβCD+2CH$_2$+NH$_4$]$^+$	1376.6540	-1.8 ppm
	1381.6065	[DMβCD+2CH$_2$+Na]$^+$	1381.6094	-2.1 ppm
	1383.5650	[DMβCD+CH$_2$+K]$^+$	1383.5677	-2.0 ppm
	1390.6665	[DMβCD+3CH$_2$+NH$_4$]$^+$	1390.6696	-2.2 ppm
	1395.6221	[DMβCD+3CH$_2$+Na]$^+$	1395.6250	-2.1 ppm
	1397.5802	[DMβCD+2CH$_2$+K]$^+$	1397.5833	-2.2 ppm
	1404.6824	[DMβCD+4CH$_2$+NH$_4$]$^+$	1404.6853	-2.1 ppm
	1493.5883	[DMβCD-CH$_2$+Cp$_2$Ti-H]$^+$	1493.5910	-1.8 ppm
	1507.6043	[DMβCD+Cp$_2$Ti-H]$^+$	1507.6067	-1.6 ppm
	1521.6197	[DMβCD+CH$_2$+Cp$_2$Ti-H]$^+$	1521.6223	-1.7 ppm
m	1535.6349	[DMβCD+2CH$_2$+Cp$_2$Ti-H]$^+$	1535.6380	-2.0 ppm
	1543.6249	[DMβCD+Cp$_2$Ti(OH)$_2$+H]$^+$	1543.6278	-1.9 ppm
	1557.6405	[DMβCD+CH$_2$+Cp$_2$Ti(OH)$_2$+H]$^+$	1557.6435	-1.9 ppm
	1571.6559	[DMβCD+2CH$_2$+Cp$_2$Ti(OH)$_2$+H]$^+$	1571.6591	-2.0 ppm

Table S21. Tandem mass spectrum of the DMβ-cyclodextrin/titanocene dichloride mixture in 50/50 H_2O/MeCN. Precursor ion: $[DM\beta CD+Cp_2Ti\text{-}H]^+$ *m/z* 1507.60, HCD at 20% NCE (54 eV), 100% = 1.18E6.

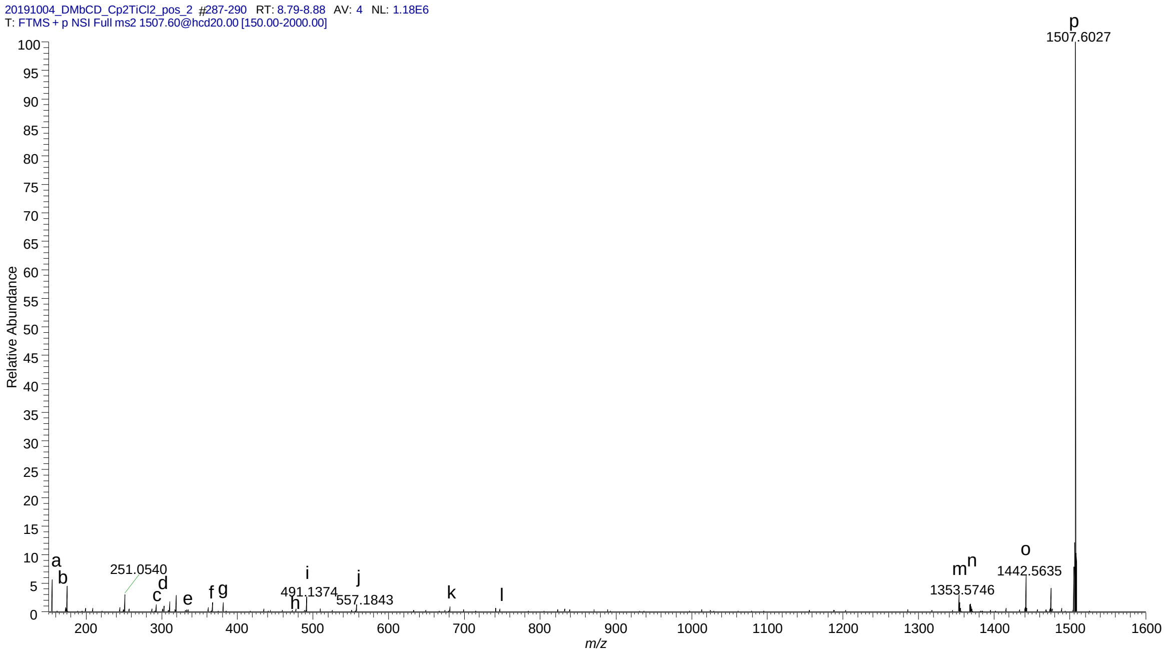

	m/z experimental	Name	*m/z* calculation	Error
a	155.0697	$[DMglc\text{-}2H_2O+H]^+$	155.0703	-3.9 ppm
b	173.0803	$[DMglc\text{-}H_2O+H]^+$	173.0808	-2.9 ppm
c	287.0386	$[DMglc\text{-}CH_2+CpTi\text{-}2H]^+$	287.0393	-2.4 ppm
d	301.0542	$[DMglc+CpTi\text{-}2H]^+$	301.0550	-2.7 ppm
e	335.0748	$[DMglc\text{-}CH_3OH+Cp_2Ti\text{-}H]^+$	335.0757	-2.7 ppm
f	367.1009	$[DMglc+Cp_2Ti\text{-}H]^+$	367.1019	-2.7 ppm
g	381.1744	$[DMglc_2+H]^+$	381.1755	-2.9 ppm
h	477.1218	$[DMglc_2\text{-}CH_2+CpTi\text{-}2H]^+$	477.1235	-3.6 ppm
i	491.1374	$[DMglc_2+CpTi\text{-}2H]^+$	491.1391	-3.5 ppm
j	557.1843	$[DMglc_2+Cp_2Ti\text{-}H]^+$	557.1861	-3.2 ppm
k	681.2214	$[DMglc_3+CpTi\text{-}2H]^+$	681.2232	-2.6 ppm
l	747.2682	$[DMglc_3+Cp_2Ti\text{-}H]^+$	747.2702	-2.7 ppm
m	1353.5746	$[DM\beta CD+Na]^+$	1353.5781	-2.6 ppm
n	1369.5496	$[DM\beta CD+K]^+$	1369.5520	-1.8 ppm
o	1442.5635	$[DM\beta CD+CpTi\text{-}H]^+$	1442.5676	-2.8 ppm
p	1507.6027	$[DM\beta CD+Cp_2Ti\text{-}H]^+$	1507.6067	-2.7 ppm

Table S22. Full scan mass spectrum of the TMβ-cyclodextrin/phenylalanine mixture in 50/50 H_2O/MeCN (100% = 7.74E7).

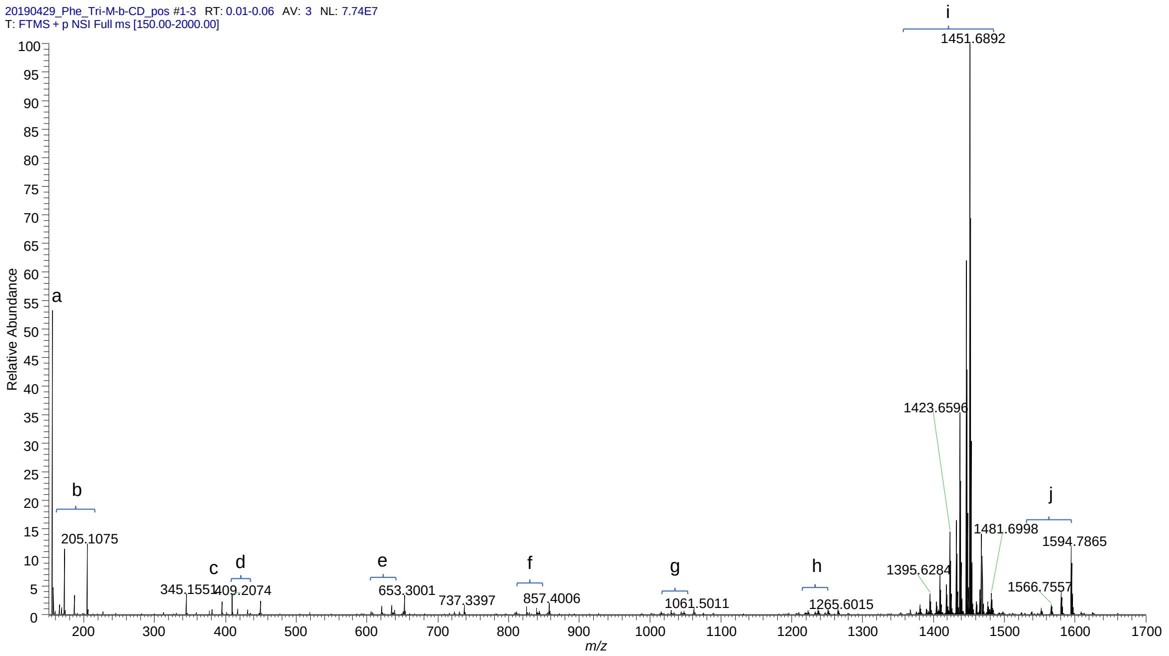

	m/z experimental	Name	*m/z* calculation	Error
a	155.0705	[TMglc-H_2O-CH_3OH+H]$^+$	155.0703	1.3 ppm
b	166.0865	[Phe+H]$^+$	166.0863	1.2 ppm
	173.0811	[TMglc-CH_3OH+H]$^+$	173.0808	1.7 ppm
	187.0968	[TMglc-H_2O+H]$^+$	187.0965	1.6 ppm
	205.1075	[TMglc+H]$^+$	205.1071	2.0 ppm
c	381.1761	[$TMglc_2$-2CH_2+H]$^+$	381.1755	1.6 ppm
	395.1918	[$TMglc_2$-CH_2+H]$^+$	395.1912	1.5 ppm
d	409.2074	[$TMglc_2$+H]$^+$	409.2068	1.5 ppm
	417.1737	[$TMglc_2$-CH_2+Na]$^+$	417.1731	1.4 ppm
	431.1894	[$TMglc_2$+Na]$^+$	431.1888	1.4 ppm
e	607.2579	[$TMglc_3$-2CH_2+Na]$^+$	607.2572	1.2 ppm
	621.2739	[$TMglc_3$-CH_2+Na]$^+$	621.2729	1.6 ppm
	635.2893	[$TMglc_3$+Na]$^+$	635.2885	1.3 ppm
f	811.3587	[$TMglc_4$-2CH_2+Na]$^+$	811.3570	2.1 ppm
	825.3743	[$TMglc_4$-CH_2+Na]$^+$	825.3727	1.9 ppm
	839.3898	[$TMglc_4$+Na]$^+$	839.3883	1.8 ppm
g	1015.4592	[$TMglc_5$-2CH_2+Na]$^+$	1015.4568	2.4 ppm
	1029.4747	[$TMglc_5$-CH_2+Na]$^+$	1029.4724	2.2 ppm
	1043.4900	[$TMglc_5$+Na]$^+$	1043.4881	1.8 ppm
h	1219.5590	[$TMglc_6$-2CH_2+Na]$^+$	1219.5566	2.0 ppm
	1233.5745	[$TMglc_6$-CH_2+Na]$^+$	1233.5722	1.9 ppm
	1247.5900	[$TMglc_6$+Na]$^+$	1247.5879	1.7 ppm
i	1353.5821	[TMβCD-7CH_2+Na]$^+$	1353.5781	3.0 ppm
	1367.5973	[TMβCD-6CH_2+Na]$^+$	1367.5937	2.6 ppm
	1376.6575	[TMβCD-5CH_2+NH_4]$^+$	1376.6540	2.5 ppm
	1381.6131	[TMβCD-5CH_2+Na]$^+$	1381.6094	2.7 ppm
	1390.6733	[TMβCD-4CH_2+NH_4]$^+$	1390.6696	2.7 ppm
	1395.6284	[TMβCD-4CH_2+Na]$^+$	1395.6250	2.4 ppm
	1404.6889	[TMβCD-3CH_2+NH_4]$^+$	1404.6853	2.6 ppm
	1409.6441	[TMβCD-3CH_2+Na]$^+$	1409.6407	2.4 ppm

	1418.7044	$[TM\beta CD-2CH_2+NH_4]^+$	1418.7009	2.5 ppm
	1423.6596	$[TM\beta CD-2CH_2+Na]^+$	1423.6563	2.3 ppm
	1425.6128	$[TM\beta CD-3CH_2+K]^+$	1425.6146	-1.3 ppm
	1432.7198	$[TM\beta CD-CH_2+NH_4]^+$	1432.7166	2.2 ppm
	1437.6748	$[TM\beta CD-CH_2+Na]^+$	1437.6720	1.9 ppm
	1439.6313	$[TM\beta CD-2CH_2+K]^+$	1439.6303	0.7 ppm
	1446.7347	$[TM\beta CD+NH_4]^+$	1446.7322	1.7 ppm
	1451.6892	$[TM\beta CD+Na]^+$	1451.6876	1.1 ppm
	1453.6483	$[TM\beta CD-CH_2+K]^+$	1453.6459	1.7 ppm
	1467.6637	$[TM\beta CD+K]^+$	1467.6616	1.4 ppm
	1524.7102	$[TM\beta CD-5CH_2+Phe+H]^+$	1524.7064	2.5 ppm
	1538.7246	$[TM\beta CD-4CH_2+Phe+H]^+$	1538.7221	1.6 ppm
	1552.7401	$[TM\beta CD-3CH_2+Phe+H]^+$	1552.7377	1.5 ppm
j	1566.7557	$[TM\beta CD-2CH_2+Phe+H]^+$	1566.7534	1.5 ppm
	1580.7709	$[TM\beta CD-CH_2+Phe+H]^+$	1580.7690	1.2 ppm
	1594.7865	$[TM\beta CD+Phe+H]^+$	1594.7847	1.1 ppm
	1597.7957	$[TM\beta CD-CH_2+Phe+NH_4]^+$	1597.7956	0.1 ppm

Table S23. Tandem mass spectrum of the TMβ-cyclodextrin/phenylalanine mixture in 50/50 H_2O/MeCN. Precursor ion: $[TM\beta CD+Phe+H]^+$ *m/z* 1594.80, HCD at 15% NCE (43 eV), 100% = 2.22E5.

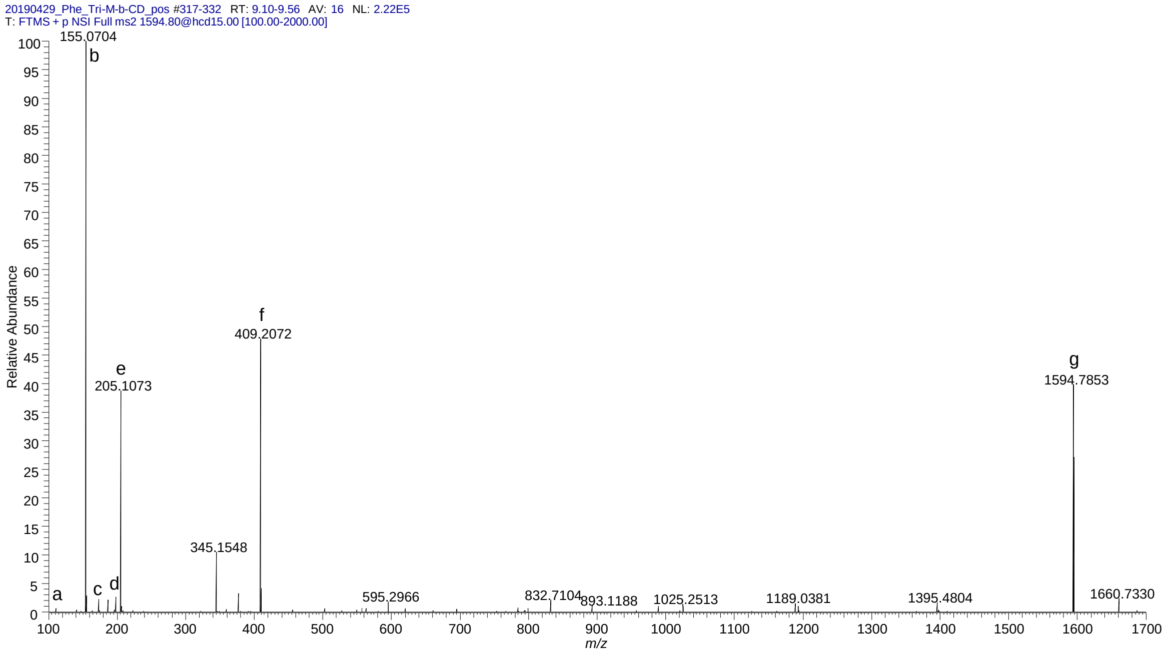

	m/z experimental	Name	*m/z* calculation	Error
a	111.0440	$[TMglc-2CH_3OH-CH_2O+H]^+$	111.0441	-0.9 ppm
b	155.0704	$[TMglc-H_2O-CH_3OH+H]^+$	155.0703	0.6 ppm
c	173.0810	$[TMglc-CH_3OH+H]^+$	173.0808	1.2 ppm
d	187.0967	$[TMglc-H_2O+H]^+$	187.0965	1.1 ppm
e	205.1073	$[TMglc+H]^+$	205.1071	1.0 ppm
f	409.2072	$[TMglc_2+H]^+$	409.2068	1.0 ppm
g	1594.7853	$[TM\beta CD+Phe+H]^+$	1594.7847	0.4 ppm

Table S24. Full scan mass spectrum of the TMβ-cyclodextrin/titanocene dichloride mixture in 50/50 H2O/MeCN (100% = 4.65E7).

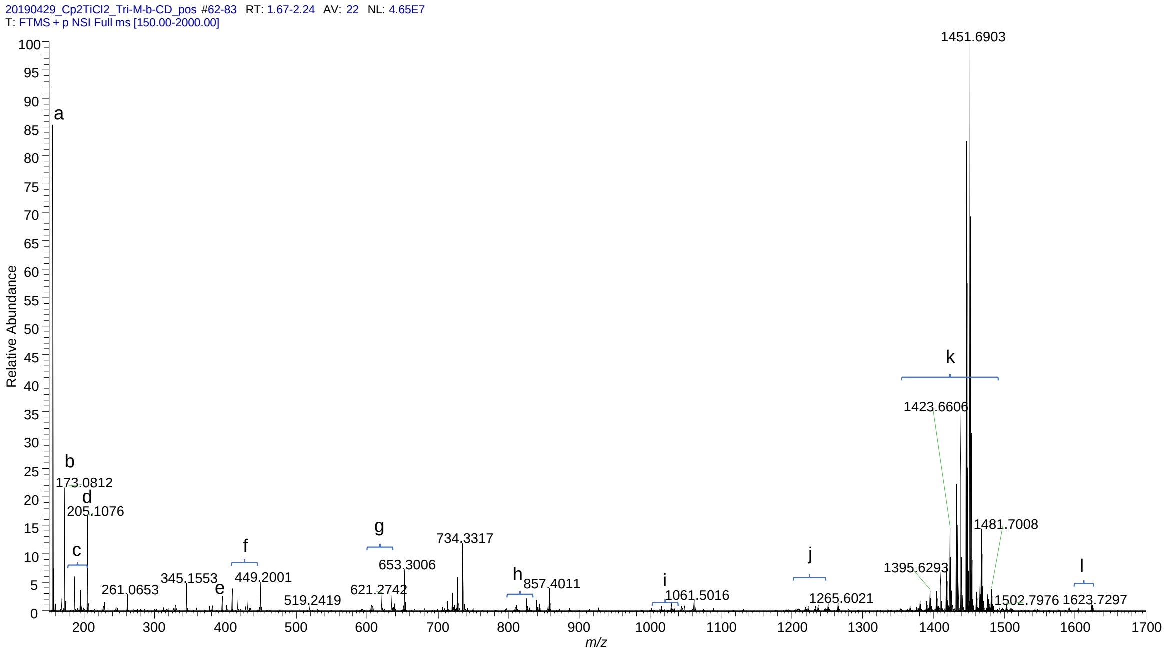

	m/z experimental	Name	*m/z* calculation	Error
a	155.0706	[TMglc-H2O-CH3OH+H]+	155.0703	1.9 ppm
b	173.0812	[TMglc-CH3OH+H]+	173.0808	2.3 ppm
c	187.0970	[TMglc-H2O+H]+	187.0965	2.7 ppm
	191.0919	[TMglc-CH2+H]+	191.0914	2.6 ppm
	195.0289	[Cp2Ti(OH)]+	195.0284	2.6 ppm
d	205.1076	[TMglc+H]+	205.1071	2.4 ppm
e	381.1764	[TMglc2-2CH2+H]+	381.1755	2.4 ppm
	395.1920	[TMglc2-CH2+H]+	395.1912	2.0 ppm
f	409.2077	[TMglc2+H]+	409.2068	2.2 ppm
	417.1740	[TMglc2-CH2+Na]+	417.1731	2.2 ppm
	431.1896	[TMglc2+Na]+	431.1888	1.9 ppm
g	607.2583	[TMglc3-2CH2+Na]+	607.2572	1.8 ppm
	621.2742	[TMglc3-CH2+Na]+	621.2729	2.1 ppm
	635.2899	[TMglc3+Na]+	635.2885	2.2 ppm
h	811.3591	[TMglc4-2CH2+Na]+	811.3570	2.6 ppm
	825.3748	[TMglc4-CH2+Na]+	825.3727	2.5 ppm
	839.3905	[TMglc4+Na]+	839.3883	2.6 ppm
i	1015.4596	[TMglc5-2CH2+Na]+	1015.4568	2.8 ppm
	1029.4754	[TMglc5-CH2+Na]+	1029.4724	2.9 ppm
	1043.4909	[TMglc5+Na]+	1043.4881	2.7 ppm
j	1205.5443	[TMglc6-3CH2+Na]+	1205.5409	2.8 ppm
	1219.5601	[TMglc6-2CH2+Na]+	1219.5566	2.9 ppm
	1233.5757	[TMglc6-CH2+Na]+	1233.5722	2.8 ppm
	1247.5910	[TMglc6+Na]+	1247.5879	2.5 ppm
k	1353.5825	[TMβCD-7CH2+Na]+	1353.5781	3.3 ppm
	1367.5980	[TMβCD-6CH2+Na]+	1367.5937	3.1 ppm
	1376.6583	[TMβCD-5CH2+NH4]+	1376.6540	3.1 ppm
	1381.6139	[TMβCD-5CH2+Na]+	1381.6094	3.3 ppm
	1390.6741	[TMβCD-4CH2+NH4]+	1390.6696	3.2 ppm
	1395.6293	[TMβCD-4CH2+Na]+	1395.6250	3.1 ppm

	1404.6898	[TMβCD-3CH$_2$+NH$_4$]$^+$	1404.6853	3.2 ppm
	1409.6450	[TMβCD-3CH$_2$+Na]$^+$	1409.6407	3.1 ppm
	1418.7052	[TMβCD-2CH$_2$+NH$_4$]$^+$	1418.7009	3.0 ppm
	1423.6606	[TMβCD-2CH$_2$+Na]$^+$	1423.6563	3.0 ppm
	1425.6143	[TMβCD-3CH$_2$+K]$^+$	1425.6146	-0.2 ppm
	1432.7207	[TMβCD-CH$_2$+NH$_4$]$^+$	1432.7166	2.9 ppm
	1437.6758	[TMβCD-CH$_2$+Na]$^+$	1437.6720	2.6 ppm
	1439.6329	[TMβCD-2CH$_2$+K]$^+$	1439.6303	1.8 ppm
	1446.7355	[TMβCD+NH$_4$]$^+$	1446.7322	2.3 ppm
	1451.6903	[TMβCD+Na]$^+$	1451.6876	1.9 ppm
	1453.6492	[TMβCD-CH$_2$+K]$^+$	1453.6459	2.3 ppm
	1467.6645	[TMβCD+K]$^+$	1467.6616	2.0 ppm
l	1577.6880	[TMβCD-2CH$_2$+Cp$_2$Ti-H]$^+$	1577.6849	2.0 ppm
	1591.7036	[TMβCD-CH$_2$+Cp$_2$Ti-H]$^+$	1591.7006	1.9 ppm
	1623.7297	[TMβCD+Cp$_2$TiOH]$^+$	1623.7268	1.8 ppm
	1641.7401	[TMβCD+Cp$_2$Ti(OH)$_2$+H]$^+$	1641.7374	1.6 ppm

Table S25. Tandem mass spectrum of the TMβ-cyclodextrin/phenylalanine mixture in 50/50 H_2O/MeCN + 1% FA. Precursor ion: [TMβCD*+Phe+H]$^+$ with a total of 19 methyl groups at *m/z* 1566.80, HCD at 15% NCE (42 eV), 100% = 3.53E5.

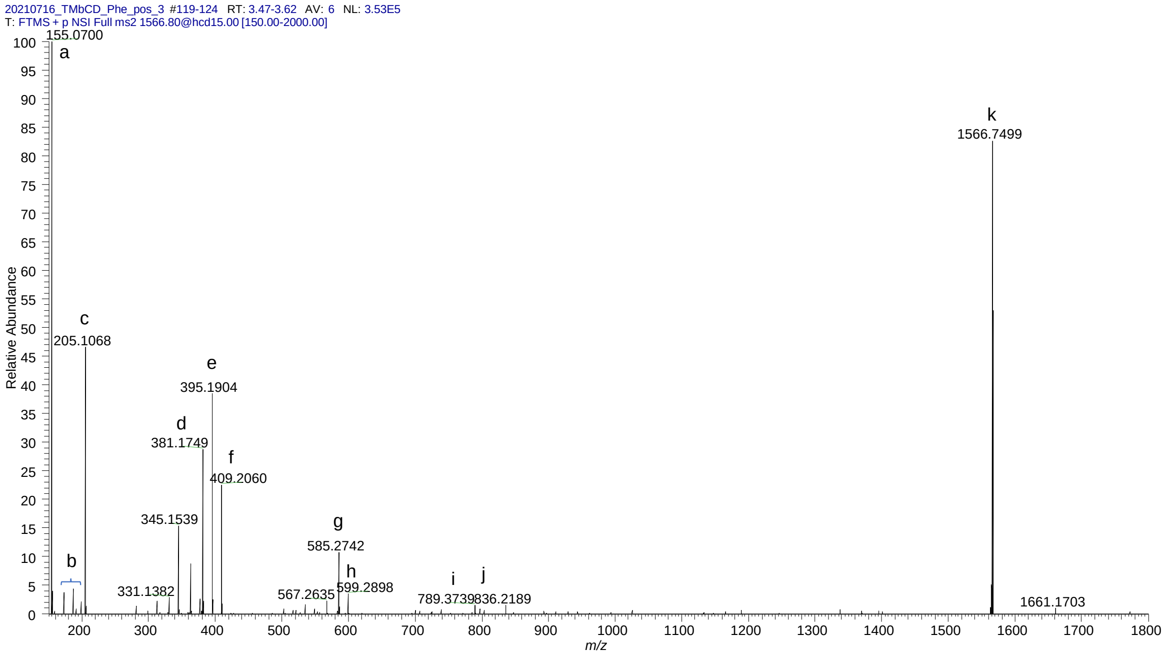

	m/z experimental	Name	*m/z* calculation	Error
a	155.0700	[TMglc-H_2O-CH_3OH+H]$^+$	155.0703	-1.9 ppm
b	173.0805	[TMglc-CH_3OH+H]$^+$	173.0808	-1.7 ppm
	187.0962	[TMglc-H_2O+H]$^+$	187.0965	-1.6 ppm
	191.0911	[TMglc-CH$_2$+H]$^+$	191.0914	-1.6 ppm
c	205.1068	[TMglc+H]$^+$	205.1071	-1.5 ppm
d	381.1749	[TMglc$_2$-2CH$_2$+H]$^+$	381.1755	-1.6 ppm
e	395.1904	[TMglc$_2$-CH$_2$+H]$^+$	395.1912	-2.0 ppm
f	409.2060	[TMglc$_2$+H]$^+$	409.2068	-2.0 ppm
g	585.2742	[TMglc$_3$-2CH$_2$+H]$^+$	585.2753	-1.9 ppm
h	599.2898	[TMglc$_3$-CH$_2$+H]$^+$	599.2909	-1.8 ppm
i	789.3739	[TMglc$_4$-2CH$_2$+H]$^+$	789.3751	-1.5 ppm
j	803.3894	[TMglc$_4$-CH$_2$+H]$^+$	803.3907	-1.6 ppm
k	1566.7499	[TMβCD-2CH$_2$+Phe+H]$^+$	1566.7534	-2.2 ppm

Table S26. Tandem mass spectrum of the TMβ-cyclodextrin/phenylalanine mixture in 50/50 H_2O/MeCN + 1% FA. Precursor ion: $[TMβCD^{\#}+Phe+H]^+$ with a total of 20 methyl groups at *m/z* 1580.80, HCD at 15% NCE (42 eV), 100% = 6.11E5.

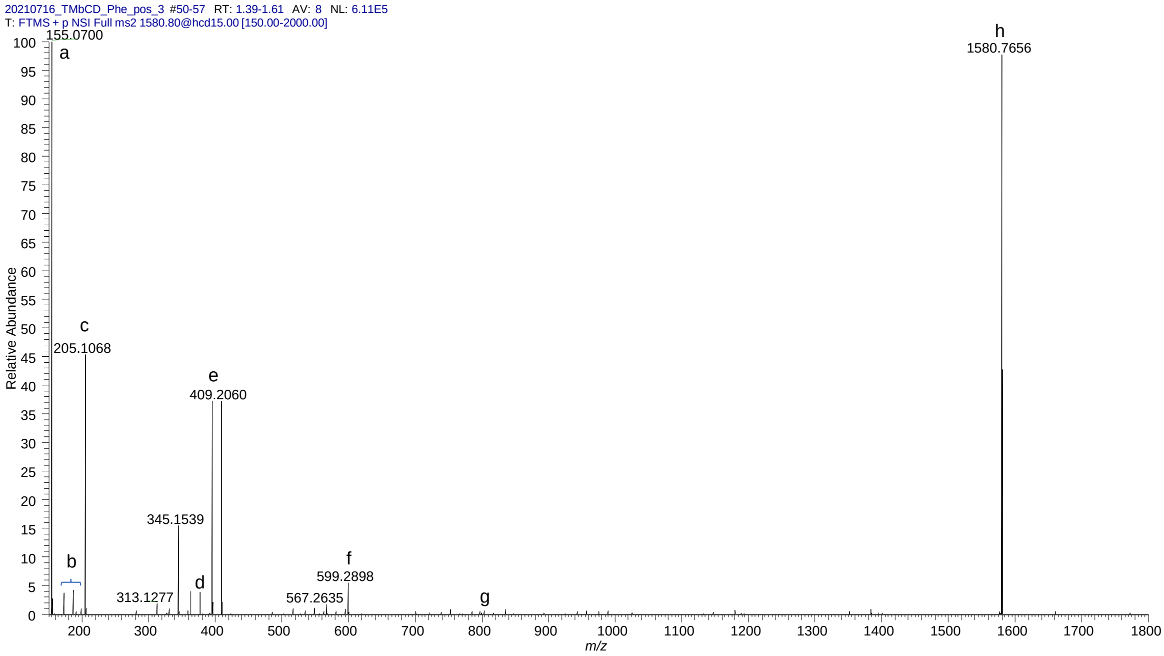

	m/z experimental	Name	*m/z* calculation	Error
a	155.07	$[TMglc-H_2O-CH_3OH+H]^+$	155.0703	-1.9 ppm
b	173.0805	$[TMglc-CH_3OH+H]^+$	173.0808	-1.7 ppm
	187.0962	$[TMglc-H_2O+H]^+$	187.0965	-1.6 ppm
	191.0911	$[TMglc-CH_2+H]^+$	191.0914	-1.6 ppm
c	205.1068	$[TMglc+H]^+$	205.1071	-1.5 ppm
d	395.1904	$[TMglc_2-CH_2+H]^+$	395.1912	-2.0 ppm
e	409.206	$[TMglc_2+H]^+$	409.2068	-2.0 ppm
f	599.2898	$[TMglc_3-CH_2+H]^+$	599.2909	-1.8 ppm
g	803.3895	$[TMglc_4-CH_2+H]^+$	803.3907	-1.5 ppm
h	1580.7656	$[TMbCD-CH_2+Phe+H]^+$	1580.7690	-2.2 ppm

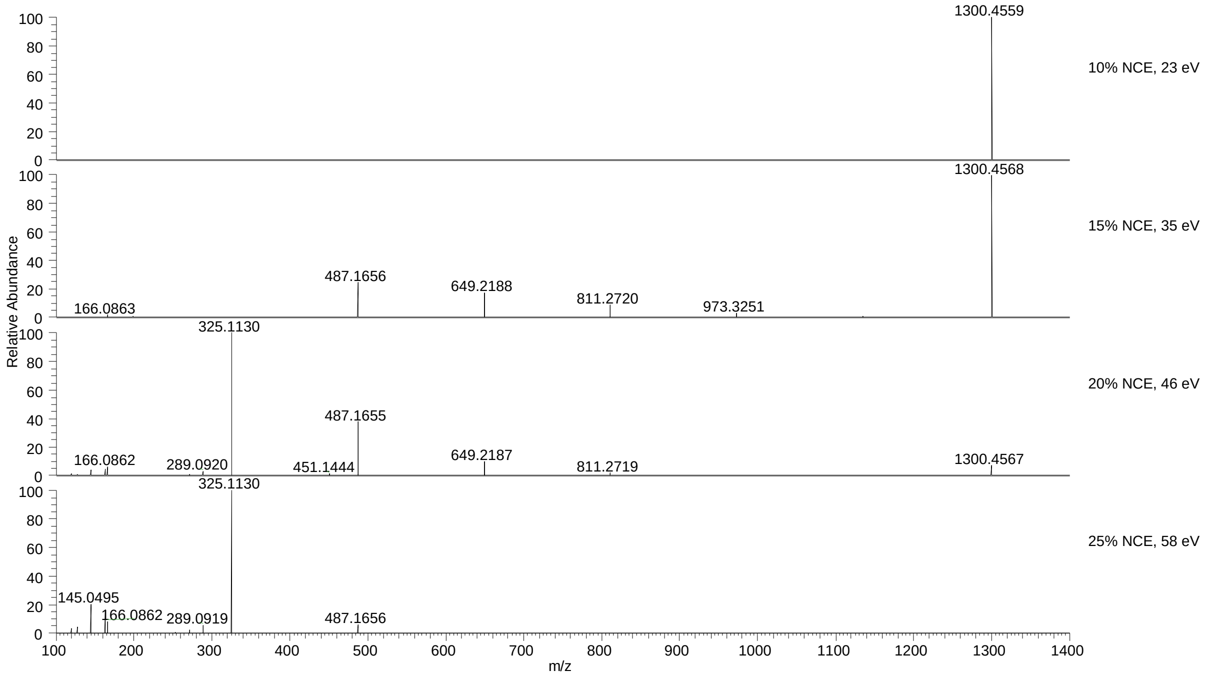

Figure S1. Tandem mass spectra of the β-cyclodextrin/phenylalanine mixture underlying the breakdown curve of [βCD+Phe+H]$^+$.

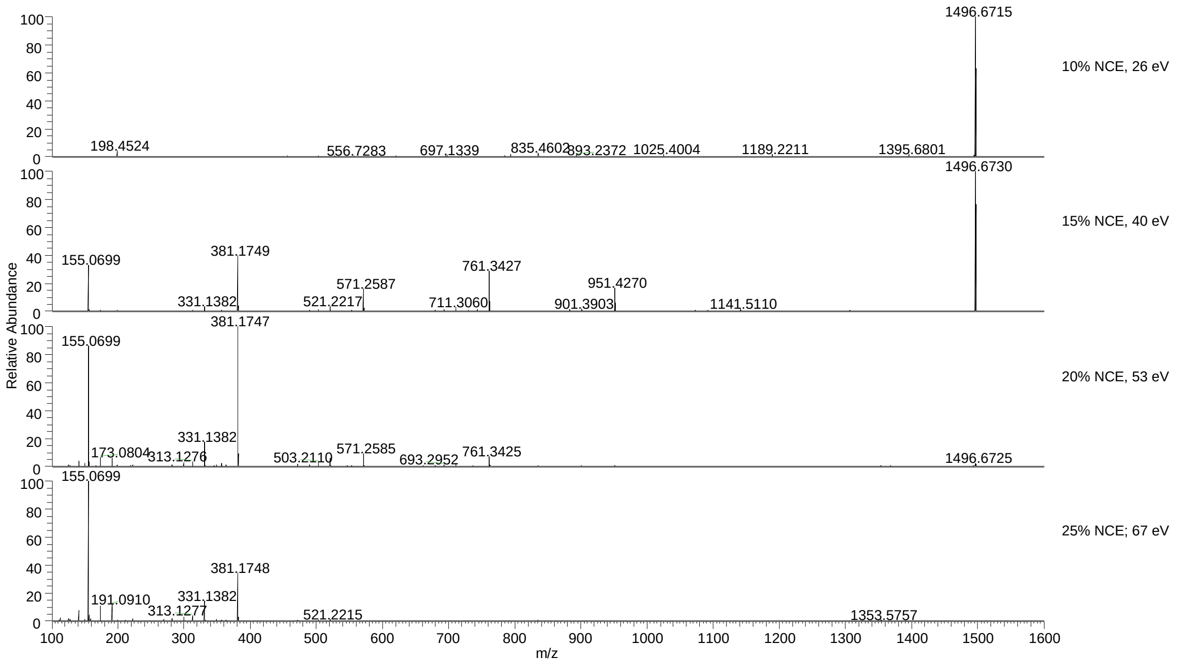

Figure S2. Tandem mass spectra of the DMβ-cyclodextrin/phenylalanine mixture underlying the breakdown curve of [DMβCD+Phe+H]$^+$.

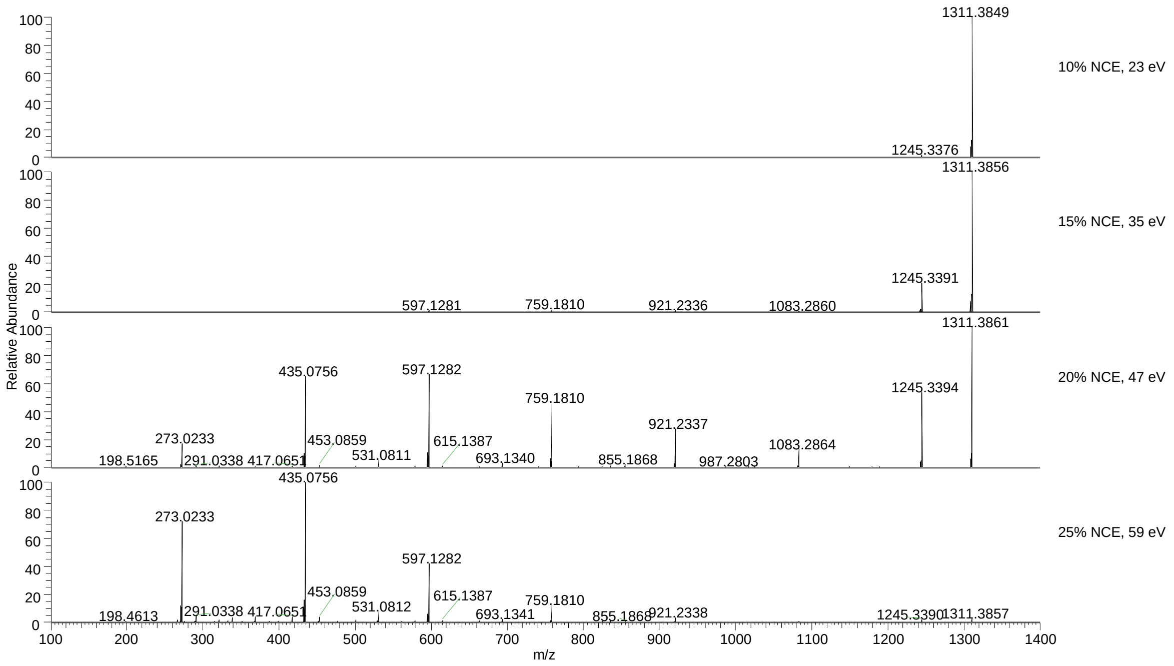

Figure S3. Tandem mass spectra of the β-cyclodextrin/titanocene dichloride mixture underlying the breakdown curve of $[\beta CD{+}Cp_2Ti{-}H]^+$.

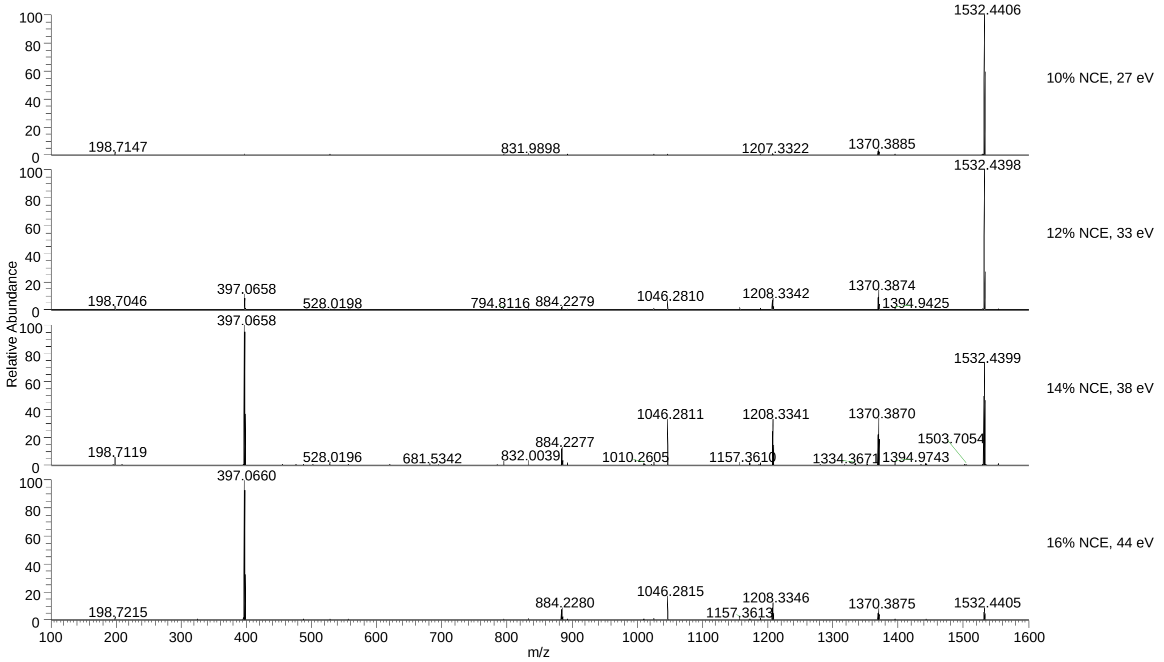

Figure S4. Tandem mass spectra of the β-cyclodextrin/oxaliplatin mixture underlying the breakdown curve of $[\beta CD{+}oxaliPt{+}H]^+$.
